# Comparative Studies on the Structural, Optical, and Electrical Properties of Two [Co(II) and Ni(II)] Complexes: Insights through Theoretical Analysis

**DOI:** 10.1021/acs.jpcb.5c02577

**Published:** 2025-07-03

**Authors:** Subhajit Saha, Samit Pramanik, Sudipta Pathak, Riya Sadhukhan, Arnab Ghosh, Dipak K. Goswami, Hon Man Lee, Rosa M. Gomila, Antonio Frontera, Subrata Mukhopadhyay

**Affiliations:** † Department of Chemistry, 30167 Jadavpur University, Kolkata 700032, India; ‡ Department of Basic Science and Humanities, Alipurduar Government Engineering and Management College, Bakla 736206, India; § Department of Chemistry, 226429 Haldia Government College, Debhog, Purba Medinipur, West Bengal 721657, India; ∥ Organic Electronics Laboratory, Department of Physics, Indian Institute of Technology Kharagpur, Kharagpur 721302, India; ⊥ Department of Physics and Natural Science Research Centre of Belda College under Vidyasagar University, Belda, West Bengal 721424, India; # Department of Chemistry, 34910 National Changhua University of Education, Changhua 50058, Taiwan; ∇ Departament de Química, 16745 Universitat de les Illes Balears, Palma, de Mallorca (Baleares) 07122, Spain

## Abstract

Two new complexes, [Co­(**N** ^**3**^ **L**)_2_]­(NO_3_)_2_·6H_2_O (complex **1**) and [Ni­(**N** ^**3**^ **L**)_2_]­(NO_3_)_2_·6H_2_O (complex **2**), have been synthesized using the organic heterocyclic chelating ligand **N** ^**3**^ **L** [4-(1-methylimidazole)-2,6-di­(pyrazinyl)­pyridine] and characterized primarily by single-crystal X-ray diffraction. In addition to detailing the crystal structures of these complexes, we highlight their distorted octahedral geometries and diverse supramolecular interactions, including π···π stacking, anion···π interactions, and hydrogen bonding. These interactions play a crucial role in shaping the distinct 1D, 2D, and 3D supramolecular architectures of both complexes. Notably, noncoordinated water molecules assemble into hexameric water clusters (H_2_O)_6_, which are key stabilizing factors for the 3D structures in the solid state. To gain deeper insight into these noncovalent interactions, we performed density functional theory (DFT) calculations combined with quantum theory of atoms in molecules (QTAIM) and noncovalent interaction plot (NCIplot) analyses. These studies allowed us to explore the nature of anion···π interactions and hydrogen bonding within the water clusters. Additionally, the electronic properties of the complexes were investigated through electrical characterization of as-fabricated Schottky diodes, revealing their potential applications in Schottky-diode-based electronic devices. Notably, in the Schottky device structure, complex **1** demonstrated superior electrical transport properties compared to complex **2** followed by its lower bandgap, better conductivity, and lower Schottky barrier height. Furthermore, we analyzed the optical properties of the Co complex (complex **1**) as a model system using band structure analysis, density of states (DOS), and projected density of states (PDOS) calculations. The superior performance of complex **1** has also been explained with proper theoretical justification.

## Graphical abstract

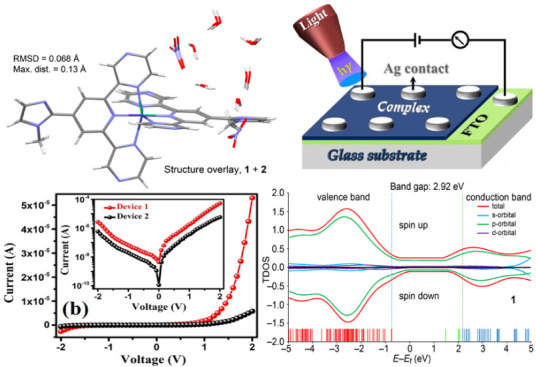

## Introduction

Supramolecular chemistry and crystal engineering, i.e., “chemistry beyond the molecule”, have developed exponentially in the past few decades because of their wide applications in different branches of science.^1^ Supramolecular science has fueled several evolutions in multidisciplinary as well as interdisciplinary domains of physics, chemistry, materials science, biology, biomedicine, etc.^2–4^ Substantial research has been carried out heretofore on supramolecular science for its important and amazing topologies. Recently, crystal engineering has gained attention in the fields of gas adsorption and its capture or storage capacity, charge-transport materials, catalysis in heterogeneous reactions, energy storage, photoluminescence, nonlinear optics, sensors, biomedicine, high-energy materials, and so on.^5–12^ Various noncovalent interactions, including hydrogen bonding, halogen bonding, van der Waals forces, π···π, cation···π, anion···π, anion···π^+^, C–H···π, and lone pair···π interactions, have been proven to have equal or even dominant contributions in explaining these properties.^13–17^

Design and synthesis of inorganic–organic hybrid versatile complex materials have received enormous attention over the past fortnight because of their potential applications in molecular adsorption,^8,18–21^ magnetism,^22–25^ electroconductivity,^26–28^ optical properties,^29,30^ and catalysis.^31–33^ Among small N-based heterocyclic organic chelating ligands, especially derivatives of terpyridine having three conjugated pyridine rings are very attractive due to their capability to create self-assembled structures and their wide range of potential applications.^32,34^ Terpyridine-based transition metal complexes have received extensive interest due to their exciting photophysical and electrochemical properties. These types of metal complexes have been employed in various research areas like optoelectronic device formation, dye-sensitized solar cells, luminescent sensor materials, and self-assembled hydrogelation.^34–41^ Thus, new analogous supramolecular synthons of terpyridine-based transition metal complexes will be designed and synthesized based on available crystallographic data and their potential applications in the literature and structural database, considering their said immense applications. Cobalt­(II) and nickel­(II) were selected for this study due to their well-characterized coordination behavior with nitrogen-donor ligands, electronic configurations conducive to tunable optical and redox properties, and their relative affordability compared to noble metals.^42–46^ These features make Co^2+^ and Ni^2+^ attractive candidates for applications in optoelectronic and magnetic materials.

Our group is currently focusing on photophysical properties of metal complexes for harvesting solar energy.^35,47,48^ In continuation of our research interest in the field of coordination chemistry and application of metal complexes, we report herein the synthesis, structural characterization, supramolecular behaviors, and photophysical properties of two new [Co­(II) and Ni­(II)] complexes. Complex **1** is formed by reacting Co­(NO_3_)_2_·6H_2_O and [4-(1-methylimidazole)-2,6-di­(pyrazinyl)­pyridine] (**N** ^**3**^ **L**) in an aqueous methanol medium. Complex **2** is synthesized by treating Ni­(NO_3_)_2_·6H_2_O with **N** ^**3**^ **L** in an aqueous acetonitrile medium. Generally, chelating ligands with nitrogen donors exert a moderately strong ligand field. Co^2+^, being a d^7^ system, commonly adopts an octahedral geometry due to its favorable electronic configuration in such an environment. Ni^2+^, a d^8^ system, can exhibit variable coordination geometries depending on the ligand field strength and steric factors. While Ni^2+^ may adopt a square planar geometry in the presence of strong-field ligands (e.g., phosphines or certain amines), in the case of nitrogen-donor ligands like those used in this study, it typically forms high-spin octahedral complexes. However, this geometry is not a result of an intrinsic preference of the Ni^2+^ ion, but rather a consequence of the spatial and electronic demands imposed by the ligand’s chelating nature. To ensure the octahedral geometry for both complexes, we strategically use the tridentate *NNN* donor in a 1:2 molar ratio so that the comparison becomes specific. In our previous works,^36^ we employed the ligand (**N** ^**3**^ **L**) for the preparation of five copper complexes with anion variation and explored their supramolecular features thoroughly. Here, we have planned to synthesize two new [Co­(II) and Ni­(II)] complexes using the same ligand and investigate their supramolecular as well as photophysical properties in a systematic way. There are a lot of driving factors responsible for tuning the structure–function correlation like metal variation, ligand variation, counteranion variation, and solvent variation. Recently, we analyzed the role of counteranions in supramolecular and photophysical behaviors of two Ni­(II) complexes derived from a 4-imidazole-2,6-di­(pyrazinyl)­pyridine ligand moiety.^47^ Here, in the present study, we purposefully restrict ourselves to analyzing the role of central metal ions in the structural as well as in the electrical properties of the title complexes, while other parameters are kept the same. Crystallographic analysis confirms that the complexes adopt the same molecularmoiety, [M­(**N** ^**3**^ **L**)_2_]­(NO_3_)_2_·6H_2_O, with the variation in their central metal ions where M = Co­(II) for complex **1** and M = Ni­(II) for complex **2**. Single-crystal X-ray analysis shows that both complexes (**1** and **2**) crystallize in a triclinic system with the space group *PT*, and their unit cell comprises two asymmetric units. It is noted that the variation in the central metal ions exhibits almost similar structural features in the present complexes. Both complexes form a similar type of 2D layer through various π···π stacking interactions. Besides π···π interactions, both complexes are stabilized by anion···π and multiple intermolecular hydrogen bonding (classical and nonclassical) interactions, which are further responsible for producing various 1D and 2D supramolecular architectures. Interestingly, the hexameric water cluster, (H_2_O)_6_, resulting from the six noncoordinated water molecules, plays a crucial role in stabilizing the solid-state structure of both complexes. Knowledge obtained from the supramolecular study and computational calculation involving water in the crystal structure may also support to scrutinize various biological incidents, such as a function of ordered H_2_O molecules inside various channels in biological systems.^49,50^ The nature of anion···π interactions and hydrogen bonding within the water clusters was analyzed by using DFT calculations, complemented by the quantum theory of atoms-in-molecules (QTAIM) and the NCI plot index as computational tools. We explored the electrical properties of both complexes. Experimentally, we assessed the potential of these synthesized complexes for application in photosensitive electronic devices, such as Schottky barrier diodes (SBDs). Besides, for the accurate determination of zero-bias Schottky barrier height, we carried out temperature-dependent *I* vs *V* measurements in the temperature range of RT (298 K) to 363 K for both devices. It was determined that complex **1**, containing a Co^2+^ ion, exhibited superior performance. This behavior was elucidated from a structural point of view as well as through DFT calculations, providing a theoretical foundation for the observed experimental results.

## Experimental Section

### Materials and Apparatus

All chemical reagents of analytical grade and solvents of spectroscopic grade were purchased from commercial suppliers and employed without further purification. Doubly freshly distilled water was utilized throughout the synthetic process, and all reactions were conducted under aerobic conditions. Elemental analyses (carbon, hydrogen, and nitrogen) were conducted using a PerkinElmer 2400 Series-II CHN analyzer, USA. Fourier transform infrared (FTIR) spectra were captured using a PerkinElmer LX-1 FT-IR spectrophotometer over the range of 4000 to 400 cm^–1^, utilizing a contemporary diamond attenuated total reflectance (ATR) accessory. The electrical characterizations of the Schottky devices were performed at room temperature (300 K) in the dark using a semiconductor parameter analyzer (Keithley 4200S) connected to source-measure units (SMUs). The ground-state absorption spectra of the respective samples (spin-coated thin films on a glass substrate) were recorded using an Avaspec-3648 UV–vis–NIR spectrophotometer.

### Synthesis of [4-(1-Methylimidazole)-2,6-di­(pyrazinyl)­pyridine] [**N^3^L**]

The ligand **N** ^**3**^ **L** was synthesized by using acetylpyrazine and 1-methyl-2-imidazolecarboxaldehyde in the presence of an aqueous NH_3_ (35%) solution, according to the reported method^36^ (Scheme 1).

**Scheme 1:**
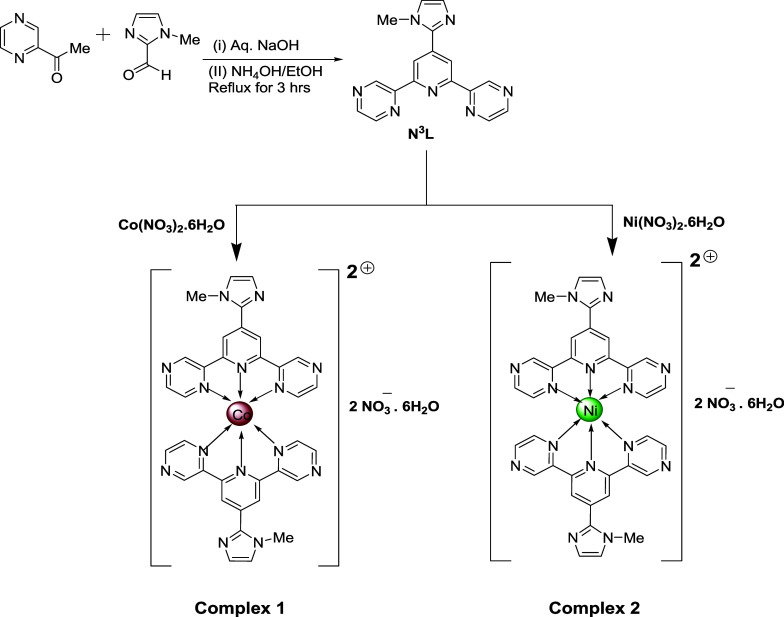
Schematic Representation of the Synthesis of the Title Complexes

### Synthesis of [Co­(N^3^L)_2_]·2NO_3_·6H_2_O (Complex **1**)

A methanolic suspension (15 mL) of the ligand, **N** ^**3**^ **L** (0.630 g, 2 mmol), was added dropwise to 15 mL of an aqueous Co­(NO_3_)_2_·6H_2_O solution (0.291 g, 1 mmol) with constant stirring at 55 °C. Then, the mixture was stirred for 3 h (Scheme 1). Then, the solution was filtered, and the filtrate was left to undergo slow evaporation without any disturbance. After 1 week, reddish-brown X-ray quality crystals of the complex were isolated (yield: 68%). Anal. calculated for C_34_H_38_N_16_CoO_12_: C, 44.3%; H, 4.12%; N, 24.32%. Found: C, 44.31%; H, 4.13%; N, 24.31%. Main FT-IR absorptions (KBr, cm^–1^): 3349 (bs), 1614 (s), 1472 (s), 1407 (vs), 1316 (s), 1141 (s), 1035 (s), 853 (s), 690 (s) (Figure S1).

### Synthesis of [Ni­(N^3^L)_2_]·2NO_3_·6H_2_O (Complex **2**)

An aqueous solution (5 mL) of Ni­(NO_3_)_2_·6H_2_O (0.290 g, 1 mmol) was added dropwise to a suspension (20 mL) of the ligand **N** ^**3**^ **L** (0.630 g, 2 mmol) in acetonitrile with constant stirring for 2 h (Scheme 1) at 60 °C. A light-green colored solution was obtained after 10 min of complete addition. The solution was then filtered, and the filtrate was kept undisturbed for crystallization. After 2 weeks, brown X-ray quality crystals of complex **1** were obtained from the mother liquor (yield: 61%). Anal. calculated for C_34_H_38_N_16_NiO_12_: C, 44.38%; H, 4.12%; N, 24.32%. Found: C, 44.39%; H, 4.12%; N, 24.33%. Main FTIR absorptions (KBr, cm^–1^): 3365.44 (bs), 1617 (vs), 1479 (vs), 1319 (vs), 1098 (s), 1037 (s), 854 (vs), 623 (vs), 416 (vs) (Figure S2).

Details of X-ray crystallographic analysis and device fabrication are provided in the .

As both complexes are nitrate salts, which are potentially explosive, we did not increase the temperature beyond 70 °C to check their thermal stability. The PXRD pattern of both complexes at room temperature (nearly 28 °C) matches well with the PXRD pattern of the complexes heated to 70 °C and cooled to room temperature. This clearly indicates the thermal stability of the complexes. Moreover, leaching experiments resulted in no change in the UV–vis spectrum of each complex even after 5 h. Also, we found that when the complexes were kept open at room temperature for 48 h, their PXRD patterns remained the same as those recorded at room temperature. All of these observations clearly indicate that both complexes and their solutions are stable against thermal decomposition or moisture adherence.

### Theoretical Methods

Interaction energies in the solid state were computed using crystallographic coordinates at the unrestricted DFT level (UPBE0-D3/def2-TZVP), with optimization applied only to hydrogen atom positions. All calculations were performed using the TURBOMOLE 7.8 software package.^51^ The PBE0 hybrid functional,^52^ which includes exact Hartree–Fock exchange, was selected due to its proven reliability in describing electronic structures and noncovalent interactions, including in Co^53–61^ and Co complexes.^62^ Grimme’s D3 dispersion correction^63^ was applied to accurately capture dispersion effects, which are particularly relevant for the supramolecular assemblies studied. The def2-TZVP basis set^64^ was employed, offering a balanced description of electron correlation and broad applicability to both main-group and transition-metal elements. No spin contamination was detected in the unrestricted calculations, confirming the reliability of the computed electronic structures.

Noncovalent interactions were characterized using the quantum theory of atoms in molecules (QTAIM)^65^ and the noncovalent interaction (NCI) index,^66^ visualized via NCIplot isosurfaces. QTAIM analysis enabled the identification of bond critical points (BCPs) and bond paths, offering a topological perspective on the interactions. NCIplot provided complementary visualization based on the reduced density gradient (RDG). The isosurface color scheme follows a red–yellow–green–blue gradient: red indicates strong repulsion, blue indicates strong attraction, and yellow to green signify weakly repulsive to weakly attractive interactions, respectively. The following NCIplot parameters were used: RDG = 0.5, electron density cutoff = 0.04 au, and a color scale range of −0.04 ≤ sign­(λ_2_)­ρ ≤ 0.04 au. Wave functions for these analyses were generated using TURBOMOLE 7.8 and processed with Multiwfn,^67^ with visualizations rendered in VMD.^68^

To explore the optical properties of the complexes, the band structure, density of states (DOS), and projected density of states (PDOS) were calculated using Multiwfn.^67^ These analyses provide insights into the electronic transitions that are relevant for potential photophysical applications.

## Results and Discussion

### Structural Description with Comparison of Complexes **1** and **2**

The asymmetric unit of complex **1** and complex **2** with the selected atom numbering scheme (only coordinated atom numbering was done for maintaining the clarity of the picture) is shown in Figures 1 and 2, respectively. Structural analysis reveals that both complexes crystallize in the triclinic system with the space group P1̅, and their unit cell comprises two asymmetric units (Table S1). For both complexes, the selected bond lengths (Å) and bond angles (°) are scheduled in Tables S2 and S3, respectively. The asymmetric unit of the title complexes contains a dicationic moiety [M­(**N** ^**3**^ **L**)_2_]^2+^, two noncoordinated nitrate ions, and six noncoordinated water molecules. Thus, the general formula for both complexes is [M­(**N** ^**3**^ **L**)_2_]­(NO_3_)_2_·6H_2_O [M = Co for complex **1** and M = Ni for complex **2**], where **N** ^**3**^ **L** [4-(1-methylimidazole)-2,6-di­(pyrazinyl)­pyridine] acts as a neutral tridentate NNN chelating ligand. The coordination mode around the metal center can be best described as a distorted octahedron with meridional geometry for both complexes. During the coordination, both complexes form four five-membered chelate rings toward the central metal ion. For complex **1**, the two pyrazinyl nitrogen atoms (N9 and N33) and the two pyridyl nitrogen atoms (N2 and N10) of our title ligand (**N** ^**3**^ **L**) generated the basal plane, and the trans axial positions are satisfied by another two pyrazinyl nitrogen atoms (N16 and N22) in the overall octahedral geometry. The average equatorial Co­(II)–N_av_ bond distance is 1.944 Å [Co1–N2 = 1.868(10) Å, Co1–N9 = 1.990(11) Å, Co1–N10 = 1.913(10) Å, and Co1–N33 = 2.004(11) Å]. The axial nitrogen atoms are positioned somewhere atlonger distances [Co1–N16 = 2.139(11) Å and Co1–N22 = 2.099(11) Å] than the equatorial bond distances [Co­(II)–N_av_ = 1.944 Å], which are in accord with previously reported six- coordinated Co­(II) complexes.^69,70^ In the octahedral geometry of complex **2**, the basal plane is formed by the two pyrazinyl nitrogen atoms (N9 and N33) and the two pyridyl nitrogen atoms (N2 and N10), where the residual *trans* axial positions are occupied by two more pyrazinyl nitrogen atoms (N16 and N22) of the title ligand (**N** ^**3**^ **L**). In the basal plane, the average equatorial Ni­(II)–N_av_ bond distance is 2.047 Å [Ni1–N2 = 1.9936(12) Å, Ni1–N9 = 2.0953(12) Å, Ni1–N10 = 1.9938(12) Å, and Ni1–N33 = 2.1058(12) Å], which is comparatively shorter than the axial bond distances [Ni1–N16 = 2.1123(12) Å and Ni1–N22 = 2.1031(12) Å]. All these Ni­(II)–N bond distances are consistent with previously reported six-coordinated Ni­(II) complexes.^47,71,72^ For both complexes, the axial bond lengths are a little bit longer than the equatorial bond lengths, likely to maintain the stereic relaxation and gain stability by lowering the symmetry. The average metal centric bond angles ∠N–M–N are 105.59° and 105.29° for complex **1** and complex **2,** respectively. In complex **1,** among the three *trans* angles, one [N2–Co1–N10 = 177.9(2)°] is close to ideal 180°, while the other two [N9–Co1–N33 = 161.4(2)° and N16–Co1–N22 = 157.1(2)°] are deviated more, maybe due to small bite angles [N10–Co1–N22 = 79.3(2)°, N10–Co1–N16 = 78.2(2)°, N2–Co1–N9 = 82.0(2)°, and N2–Co1–N33 = 79.8(2)°] of the tridentate ligand. A similar situation (as that of complex **1**) is witnessed for complex **2,** having different *trans* angles [N2–Ni1–N10 = 177.57(5)°, N9–Ni1–N33 = 155.79(5)°, and N16–Ni1–N22 = 155.89(5)°] and bite angles [N10–Ni1–N22 = 78.03(5)°, N10–Ni1–N16 = 78.10(5)°, N2–Ni1–N9 = 78.32(5)°, and N2–Ni1–N33 = 77.81(5)°]. These small bite angles are possibly responsible to induce a distortion in the *trans* angles and in the overall octahedral geometry for both complexes. In complex **1**, the Co­(II) ion is shifted by a distance of 0.018 Å toward the axial nitrogen atom (N22) from the basal plane (N2N9N10N33), whereas in complex **2**, the Ni­(II) ion is deviated by 0.023 Å toward the axial N22 atom from the basal plane (N2N9N10N33). The dipositive charges of both title complexes are taken care of by two noncoordinated nitrate ions present outside the metal coordination sphere.

**Figure 1:**
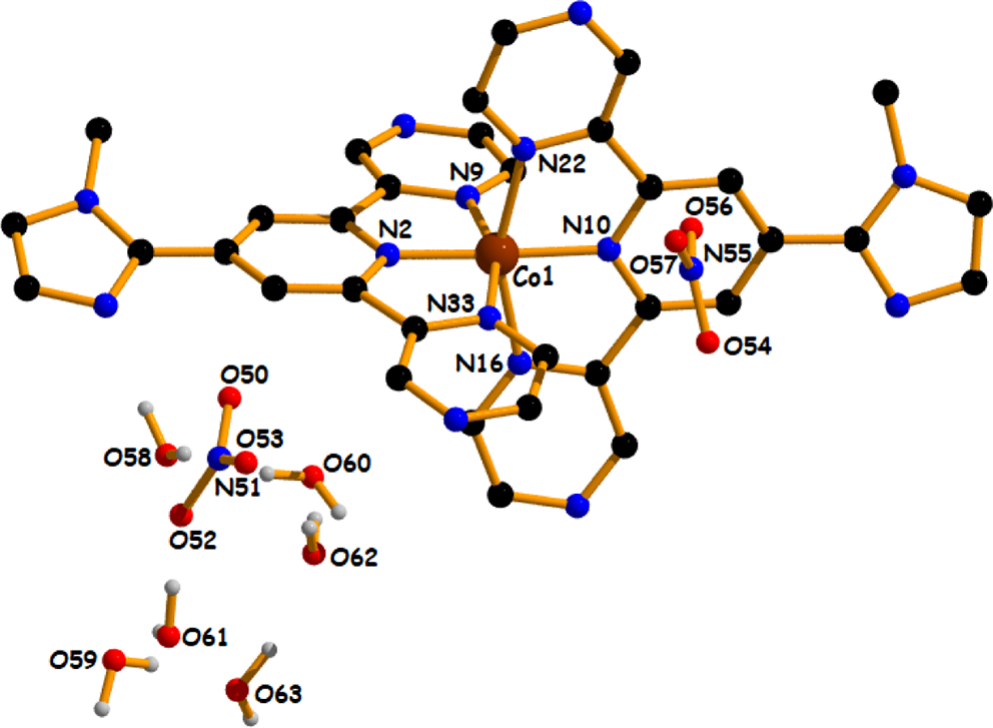
Asymmetric unit of complex **1** (aromatic hydrogen atoms have been omitted for maintaining the clarity of the picture).

**Figure 2:**
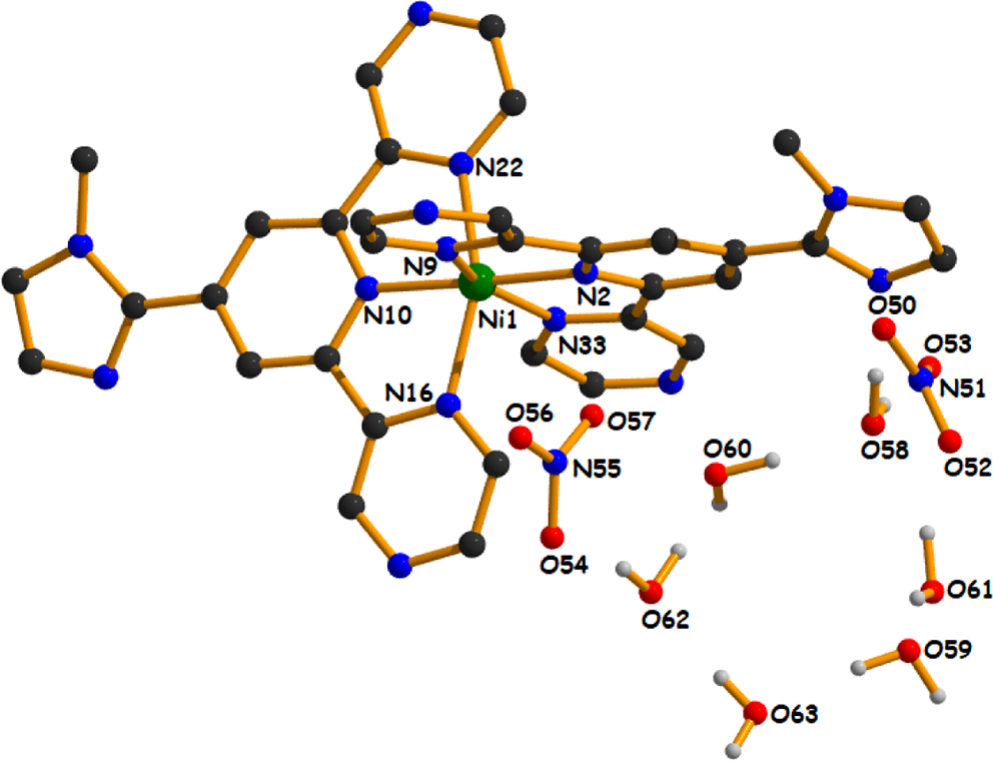
Asymmetric unit of complex **2** (aromatic hydrogen atoms have been omitted for maintaining the clarity of the picture).

It is also noteworthy to mention that the M–N_pyridyl_ bond lengths are, to some extent, shorter than those of the M–N_pyrazinyl_ bond lengths for both complexes, which may be due to the stronger π-accepting properties of the central pyridine ring or the constricted coordination geometry imposed by the ligand moiety. Here, in the present work, the title ligand (**N** ^**3**^ **L**) utilizes two pyrazinyl and one pyridyl nitrogen atoms in the coordination game. Through this coordination, the aromatic rings become electron-deficient and make them suitable for anion···π interaction. Besides, the methyl substitution in the pendant imidazole ring makes the ring twisted from the plane of the central pyridine ring. Thus, we may conclude that the central pyridine and two substituted pyrazine rings became more electropositive in character (as they are directly involved in metal coordination) compared to the peripheral substituted imidazole ring (which is involved in stronger hydrogen bonding interactions compared to the pyridine and pyrazine rings).

The solid-state structure of both complexes is stabilized through a combination of π···π, anion···π, and numerous hydrogen-bonding interactions (Tables S4, S5, and S6), which are crucially responsible for the formation of various supramolecular architectures (1D, 2D, and 3D). In complex **1**, the dicationic moiety [Co­(**N** ^**3**^ **L**)_2_]^2+^ of the asymmetric unit propagates through two different face-to-face π···π interactions [Cg(5)···Cg(5) at (2 – *x*, −*y*, 2 – *z*) and Cg(6)···Cg(6) at (−*x*, 1 – *y*, 1 – *z*)], which repeat alternately to ensure a 1D polymeric association (Figure 3). This 1D arrangement is further extended to 2D layers using another two different self-complementary π···π interactions [Cg(5)···Cg(10) at (1 – x, −*y*, 2 – *z*) and Cg(6)···Cg(12) at (1 – *x*, 1 – *y*, 1 – *z*)], as depicted in Figure 3. During the formation of this 2D layer, the dicationic moieties are arranged almost in opposite orientations, where the average Cg···Cg separation is 3.594 Å. For better understanding, we have colored the rings as yellow for Cg(5) [centroid of the (N36/C35/C34/N38/C37) ring], pink for Cg(6) [centroid of the (N46/C45/C44/N48/C47) ring], blue for Cg(10) [centroid of the (N16/C17/C18/N19/C20/C21) ring], and green for Cg(12) [centroid of the (N30/C29/C28/N33/C32/C31) ring].

**Figure 3:**
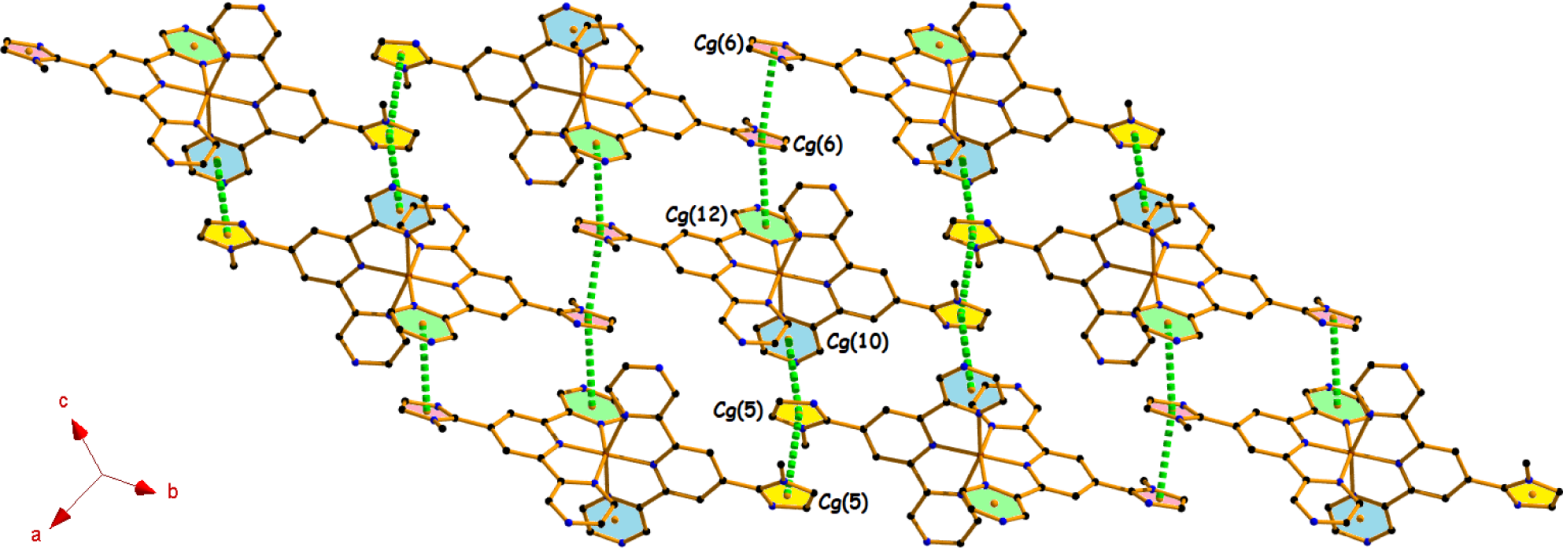
Perspective view of 2D architecture incorporating π···π interactions in complex **1** (hydrogen atoms have been omitted for the sake of clarity).

A very similar architecture (as that of Figure 3) is found for complex **2**, involving the π···π interactions [Cg(5)···Cg(5), Cg(5)···Cg(10), Cg(6)···Cg(6), and Cg(6)···Cg(12)], having similar symmetry with an average interplanar spacing of 3.569 Å (Figure S3).

Interestingly, the six noncoordinated water molecules interact among themselves to decorate a hexameric water cluster in both complexes. The water cluster is represented as ring-like, chair-like, and twist-boat-like with the atom numbering scheme (Figure 4). In complex **1**, the water cluster is formed through six O–H···O hydrogen bonding interactions [O59–H59B···O63, O60–H60B···O58, O61–H61A···O59, O61–H61B···O58, O62–H62A···O60, and ···63–H63A···O62], where the average H···O distance is 1.976 Å and the average O–H···O angle is 162.33°. Here, the noncoordinated water oxygen atom O61 acts as a double donor to the oxygen atoms (O58 and O59), and O58 acts as a double acceptor of two hydrogen atoms (H60B and H61B) from the adjacent noncoordinated water molecules, whereas the remaining noncoordinated water molecules (O59, O60, O62, and O63) act both as single donors and single acceptors in the hydrogen bonding interactions, thus forming an R_6_^6^(12) ring motif (Figure 4).

**Figure 4:**
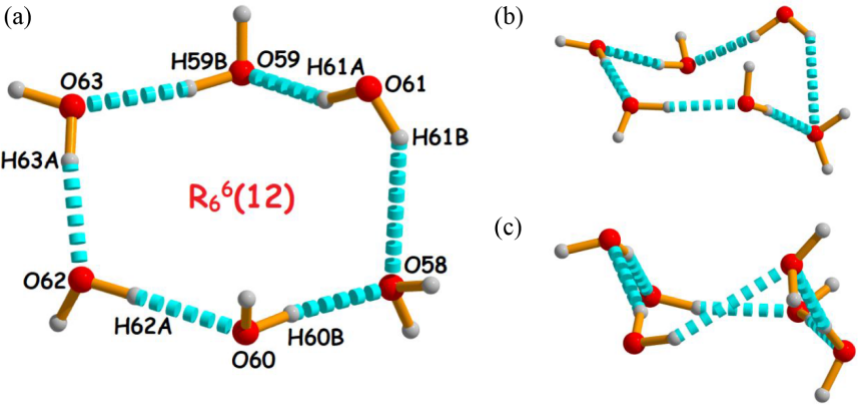
Perspective view of the hexameric water cluster with atom-numbering scheme formed in complex **1** and graphical representations of the water cluster as (a) ring-like, (b) chair-like, and (c) twist-boat-like.

A very similar water cluster (as in complex **1**) is formed in complex **2,** involving the following hydrogen bonding interactions [O59–H59B···O63, O60–H60B···O58, O61–H61A···O59, O61–H61B···O58, O62–H62A···O60, and O63–H63A···O62], with the average H···O distance and the average O–H···O angle being 1.93 Å and 162.17°, respectively (Figure S4). Here, the noncoordinated water molecules interact with each other in a similar fashion to that of complex **1,** with different H···O distances and O–H···O angles. It is well established that a lower H···A separation and a D–H···A angle close to 180° indicate a stronger hydrogen bonding interactions. It is worth mentioning that the average O···O separation distances within the hexamer are 2.803 Å (complex 1) and 2.815 Å (complex 2), which are slightly longer than the values in ice *I*_c_ (2.75 Å) and ice *I*_h_ (2.759 Å) but somewhat shorter than the value observed in liquid water (2.854 Å). Besides, the average O–O–O bond angles are 103.09° (for complex **1**) and 105.98° (for complex **2**), which are close to the tetrahedral angle in ice *I*_h_ form.^37^

The hexameric water cluster is stabilized through the combination of various classical (O–H···O and O–H···N) and nonclassical (C–H···O) hydrogen bonding interactions in both complexes. In complex **1**, the dicationic moiety firmly holds the water cluster through four different hydrogen bonding interactions [C40–H40···O61 (149°), C41–H41···O59 (140°), C18–H18···O62 (134°), and O58–H58A**···**N46 (175°)], where the first two interactions are responsible for the formation of an R_3_^2^(7) ring motif (Figure 5). These interactions are repeated among themselves, resulting in a 1D polymeric chain along the (010) direction, as depicted in Figure 5.

**Figure 5:**
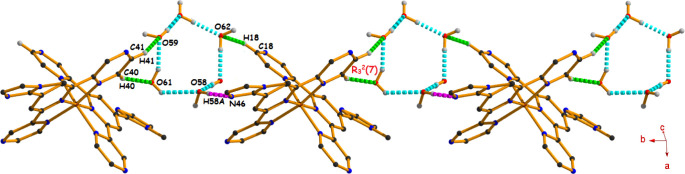
1D polymeric chain formation combining C–H···O (shown by green dotted lines), O–H···O (shown by cyan dotted lines), and O–H···N (shown by pink dotted lines) hydrogen bonding interactions in complex **1** (other hydrogen atoms have been omitted for clarity).

However, in the 1-D polymeric chain of complex **2**, the water cluster is stabilized only by three hydrogen bonds [C40–H40···O61 (156°), C18–H18···O62 (137°), and O58–H58A···N46 (179°)] with different angles of interaction (Figure S5).

Water molecules, in addition to forming clusters among themselves, also interact with nitrate anions that participate in the assembly process to maintain a donor–acceptor balance, leading to the stabilization of the overall solid-state structure for both complexes.

Here, nitrate ions play a crucial role in the stabilization of not only the water cluster but also the self-assembled structures of both complexes. In Figure 6, we have shown how the nitrate anions and the water cluster interplay among themselves to form a supramolecular association (Figure 6) through anion···π and hydrogen bonding interactions. Between two nitrate anions, one nitrate anion (N55) interacts simultaneously with Cg(2) and Cg(7) through the O57 atom, having the same symmetry (1 + *x*, *y*, *z*), while the other anion (N51–O50) interacts only with Cg(7) at (1 – *x*, 1 – *y*, 1 – *z*) of the dicationic moiety to produce the anion···π interactions in a self-complementary fashion. Now, these two anions stabilize the water cluster through four different self-complementary hydrogen bonding interactions [O58–H58B**···**O50 (154°), O58–H58B···O52 (145°), O61–H61B···O52 (143°), and O62–H62B···O54 (163°)].

**Figure 6:**
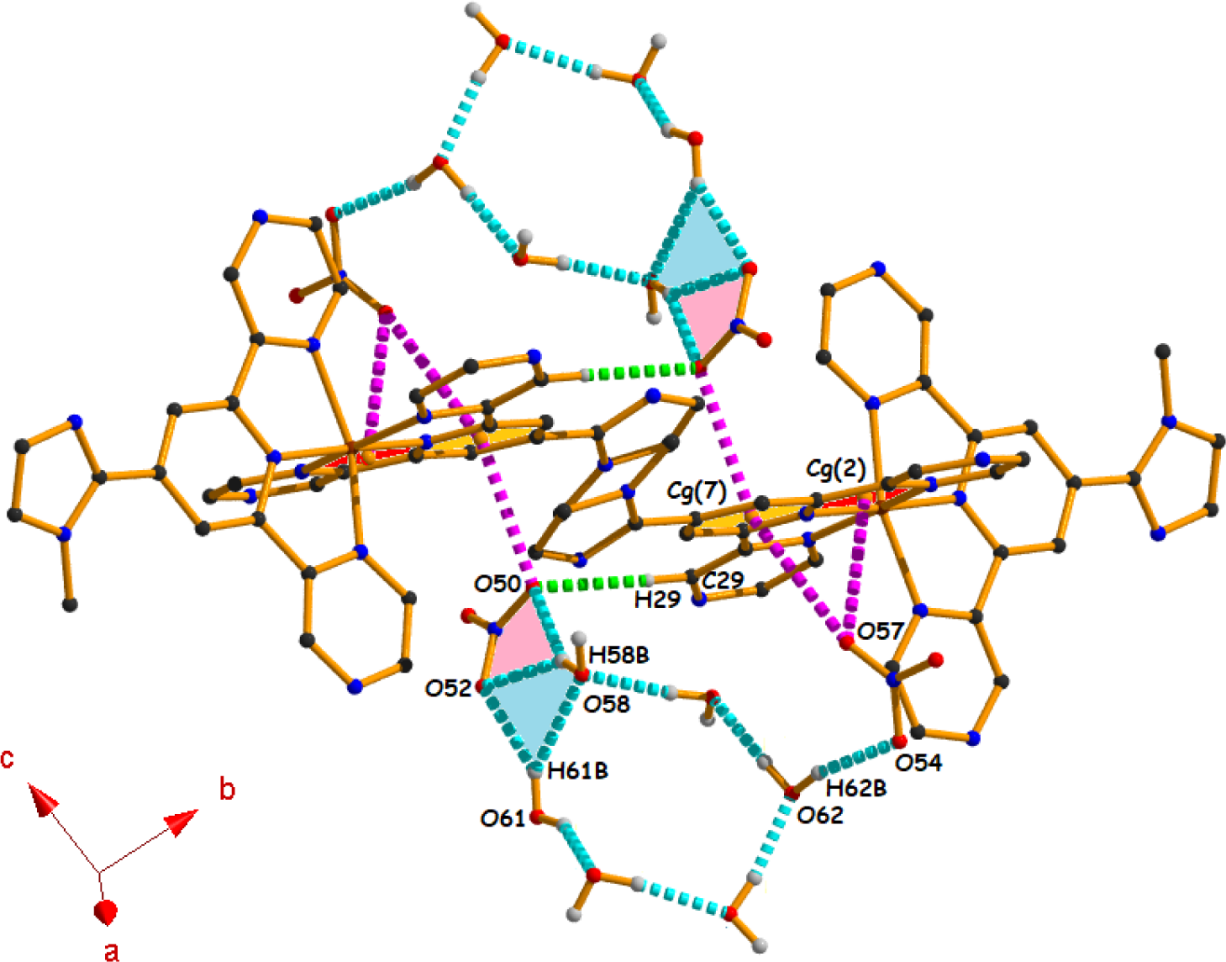
Supramolecular association involving anion···π (shown by pink dotted lines) and hydrogen bonding interactions in complex **1** (other hydrogen atoms have been omitted for clarity).

Through a closer look, it is evident that the first three hydrogen bonding interactions produce two different four-membered rings with R_1_^2^(4) [filled with pink color] and R_2_^2^(4) [filled with blue color] ring motifs. The association is further strengthened by other self-complementary C29–H29···O50 (169°) bonding interactions (shown by green dotted lines). For better understanding, we have shown the rings as red for Cg(2) [centroid of the (Co1/N2/C7/C8/N9) ring] and golden yellow for Cg(7) [centroid of the (N2/C3/C4/C5/C6/C7) ring].

In the case of complex **2**, the nitrate anions interact with the dicationic moieties and the water cluster in a similar fashion through anion···π [N51–O50···Cg(7) at (1 – *x*, 1 – *y*, 1 – *z*), N55–O57···Cg(2) at (*x*, *y*, *z*) and N55–O57···Cg(7) at (*x*, *y*, *z*)] and five self-complementary hydrogen bonding interactions [O58–H58B···O50 (154°), O58–H58B···O52 (145°), O61–H61B···O52 (143°), O62–H62B···O54 (163°), and C29–H29···O50 (169°)] to generate the supramolecular assembly (Figure S6). Upon comprehensive analysis, it is found that the average Cg···O distances are 3.264 Å (for complex **1**) and 3.270 Å (for complex **2**), leading to the comparatively stronger anion···π interaction for complex **1**.

Besides, due to the self-complementary nature of complex **1**, the dicationic moiety [Co­(**N** ^**3**^ **L**)_2_]^2+^ forms another supramolecular assembly (Figure 7), where the water cluster is stabilized by hydrogen bonds. Here, the water cluster connects the dicationic moiety through the combination of three different self-complementary hydrogen-bonding interactions [C17–H17···O57 (131°), C20–H20···O61 (168°), and O62–H62B···O54 (163°)].

**Figure 7:**
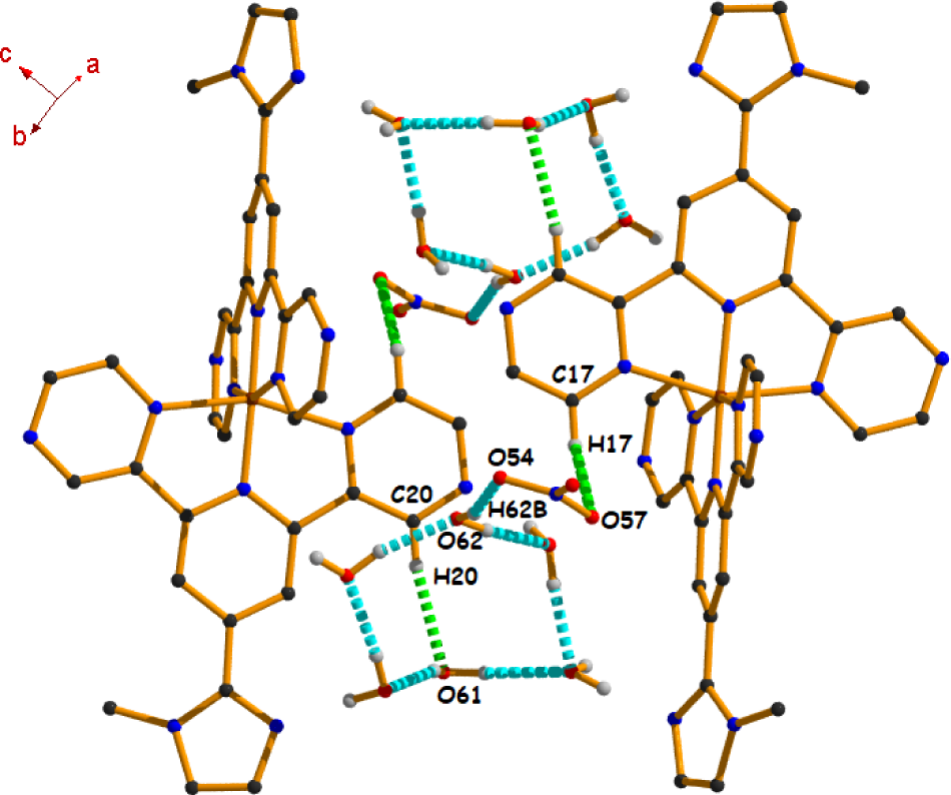
View of dimeric assembly formed by C–H···O and O–H···O hydrogen bonding interactions in complex **1** (other hydrogen atoms have been omitted for clarity).

In complex **2**, the water cluster is stabilized in a similar way (as in complex **1**), involving three hydrogen bonding interactions [C17–H17···O57(132°), C20–H20···O61 (169°), and O62–H62B···O54 (169°)] having different angles of interaction (Figure S7).

In addition, a 1D tape-like architecture is formed, involving only C–H···O hydrogen bonding interactions in complex **1** (Figure 8). Here, the dicationic moieties and the nitrate anions are arranged in a self-complementary fashion and interlink among themselves through six different hydrogen bonding interactions [C23–H23···O50 (161°), C26–H26···O53 (143°), C34–H34···O54 (143°), C39–H39A···O52 (141°), C44–H44···O57 (124°), and C49–H49B···O53 (166°)] that lead to the formation of an R_8_^8^(32) ring motif.

**Figure 8:**
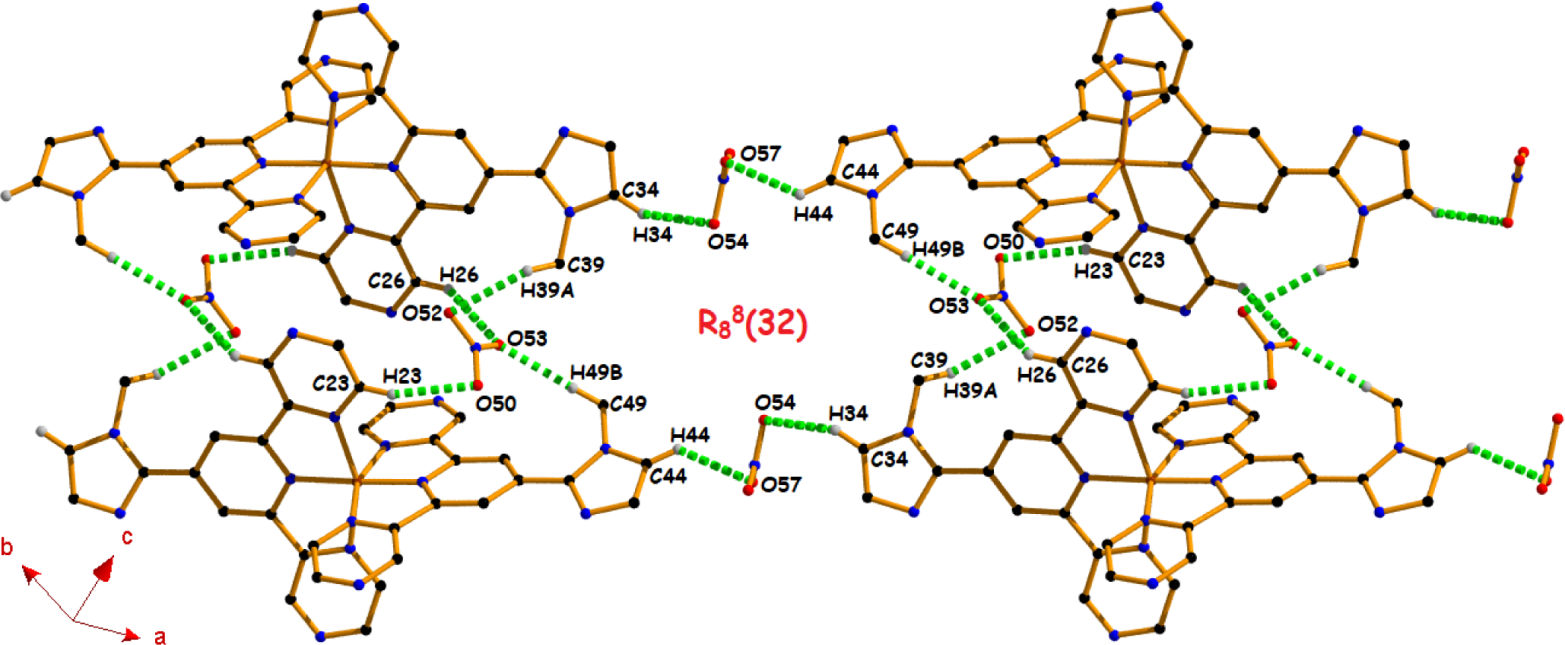
Formation of 1D tape-like architecture through C–H···O hydrogen bonding interactions in complex **1** (other hydrogen atoms have been omitted for clarity).

A very similar architecture (as that of Figure 8) is formed in complex **2** through the six self-complementary hydrogen bonding interactions [C23–H23···O50 (162°), C26–H26···O53 (144°), C34–H34···O54 (144°), C39–H39A···O52 (144°), C44–H44···O57 (124°), and C49–H49B···O53 (170°)], resulting in the same ring motif, R_8_^8^(32) (Figure S8).

From the structural elucidation, we may conclude that even after variation in central metal ions (Co^2+^ and Ni^2+^), the basic stereochemical properties remain almost the same, and this is reflected in their virtually comparable crystal packing parameters. From the supramolecular point of view, it may also be concluded that the nature of interactions is almost similar despite differences in geometrical parameters of both complexes.

### DFT Calculations

Previous theoretical studies on similar systems have demonstrated that these π-extended ligands, whether coordinated to metal centers or protonated, provide an extended electron-deficient π-surface that facilitates strong anion−π interactions.^35,47,54^ In some cases, these interactions are complemented by CH···anion contacts, as observed in this study (see Figures 6–8). Theoretical frameworks have already been established for anions such as Cl^–^, ClO_4_^–^, NO_3_^–^, and [MCl_4_]^2–^ (M = Zn, Cd, and Hg), providing valuable insights into the role of such interactions in supramolecular assembly.

For the analysis of the supramolecular structures, we focused on a single compound, as the X-ray packings of both crystal structures are nearly identical. Figure 9 presents the structural overlay of both compounds, revealing a very low root-mean-square deviation (RMSD) of 0.068 Å, with a maximum deviation of only 0.13 Å between equivalent atoms. This confirms that the two structures are essentially identical. To optimize computational efficiency, we selected compound **2** due to its lower number of unpaired electrons for the DFT analysis of noncovalent interactions.

**Figure 9:**
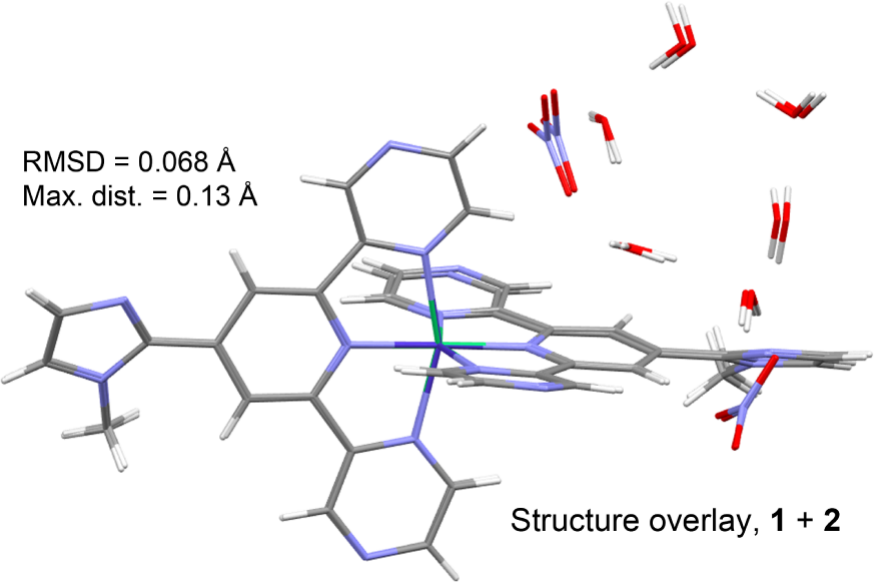
Overlay of the structures of compounds **1** and **2** with indication of the RMSD and maximum distance.

We first investigated the anion−π interactions both energetically and through a combination of QTAIM and NCI plot analyses, as these methods are particularly effective for visualizing noncovalent interactions in real space. The QTAIM analysis reveals a complex interplay of interactions, where the anions are linked to the Ni-coordinated pyridine ring via three bond critical points (BCPs, represented as red spheres) and bond paths (represented as orange lines) positioned above and below the ring. Additionally, the anions establish CH···O contacts with the pyrazine rings, each characterized by a corresponding BCP and bond path. Notably, the nitrate anion located above the pyridine ring in Figure 10 is also connected to a carbon atom of the pyrazine ring by a BCP and bond path, confirming the presence of an anion−π interaction involving a specific C atom of the ring.

**Figure 10:**
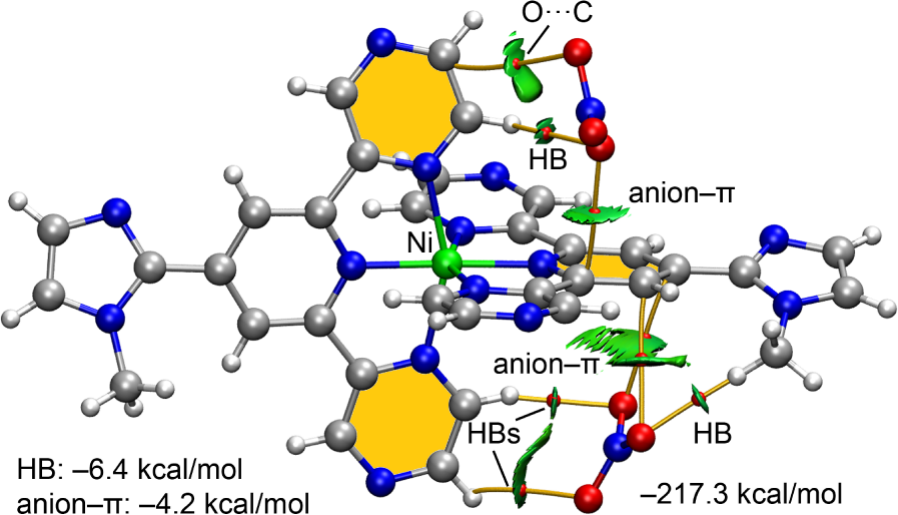
Combined QTAIM (BCPs as red spheres and bond paths as orange lines) and NCIPlot (RDG = 0.5, ρ_cutoff_ = 0.04 au, color scale ±0.035 au) analyses of the trimeric assembly of compound **2**. Only intermolecular interactions are indicated. The formation energy is indicated along with the estimations of HB and anion−π interactions using the potential energy densities of the BCPs.

The NCIplot analysis further supports the existence of hydrogen bonds and anion−π interactions. In all cases, the interactions are characterized by green reduced density gradient (RDG) isosurfaces, indicative of moderate interaction strength (the NCIplot for the Co complex is represented in the Supporting Information, showing almost identical isosurfaces; see Figure S9). The π-character of the anion−π interactions is particularly well represented by the RDG isosurfaces, which span the space between the interacting oxygen atom and the π-system of the pyridine ring. The overall interaction energy of this assembly is substantial (−217 kcal/mol), primarily reflecting the dominant electrostatic character of the ion-pair interaction. This value corresponds to the difference between the total energy of the assembly and the sum of the energies of the isolated monomers, namely, two nitrate anions and the [Co­(N_3_L)_2_]^2+^ complex. The interaction energy has been corrected for basis set superposition error (BSSE).^73,74^

To obtain a more accurate estimate of the interaction energies, we employed QTAIM parameters evaluated at the bond critical points (BCPs) and applied an energy estimation method based on the potential energy density, as proposed by Espinosa et al.^73^ Specifically, the interaction energies were estimated using the expression *E* = 0.5 *V*, where *V* is the potential energy density at the BCP. Using this approach, the combined interaction energy of the CH···O hydrogen bonds is −6.4 kcal/mol, while the total contribution of the anion−π interactions is −4.2 kcal/mol. These results confirm the modest interaction strengths suggested by the NCIplot analysis and further indicate that hydrogen bonding plays a slightly more significant role than anion−π interactions in stabilizing the assembly.

Second, we analyzed the hexameric water cluster and its role in bridging the metal complexes and nitrate anions. Figure 11 presents the QTAIM analysis of the water cluster, highlighting its interaction with two [Ni­(**N** ^**3**^ **L**)_2_]^2+^ units through OH···N hydrogen bonds. The formation energy of the hexameric water cluster in the presence of the [Ni­(**N** ^**3**^ **L**)_2_]^2+^ units is notably high (−48.3 kcal/mol), with each hydrogen bond contributing approximately −8.0 kcal/mol.

**Figure 11:**
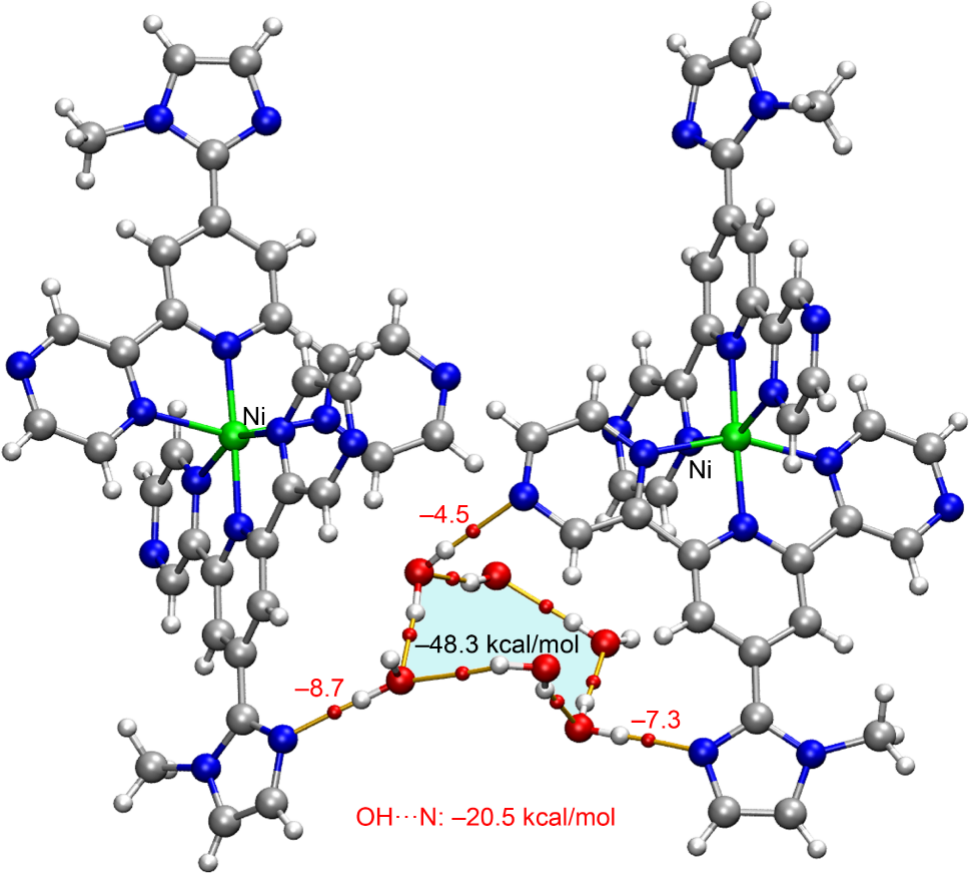
QTAIM analysis (BCPs as red spheres and bond paths as orange lines) of the hexameric water cluster in the presence of two [Ni­(**N** ^**3**^ **L**)_2_]^2+^ units. The energies of the OH···N H-bonds are indicated in red adjacent to the BCPs.

Notably, the OH···N (methylimidazole) hydrogen bonds are stronger than the OH···N (pyrazine) interactions. This difference is likely due to the coordination of the opposite nitrogen atom of pyrazine to the Ni metal center, which reduces the lone-pair donor ability of the uncoordinated nitrogen. This observation underscores the subtle electronic effects introduced by metal coordination on hydrogen bond strength and overall cluster stability.

The QTAIM analysis of the assembly, where the hexameric water cluster bridges the anions, is depicted in Figure 12. In this case, the water cluster exhibits a slightly more negative formation energy (−48.5 kcal/mol), suggesting a slightly enhanced cooperativity effect for the OH···O (nitrate) hydrogen bonds compared to the OH···N interactions. Two of the nitrate anions are connected to the hexameric cluster through a single BCP and bond path, while a third anion establishes a bifurcated OH···O_2_ hydrogen bond (−8.5 kcal/mol in total), along with an additional OH···O hydrogen bond with a separate water molecule (−3.6 kcal/mol).

**Figure 12:**
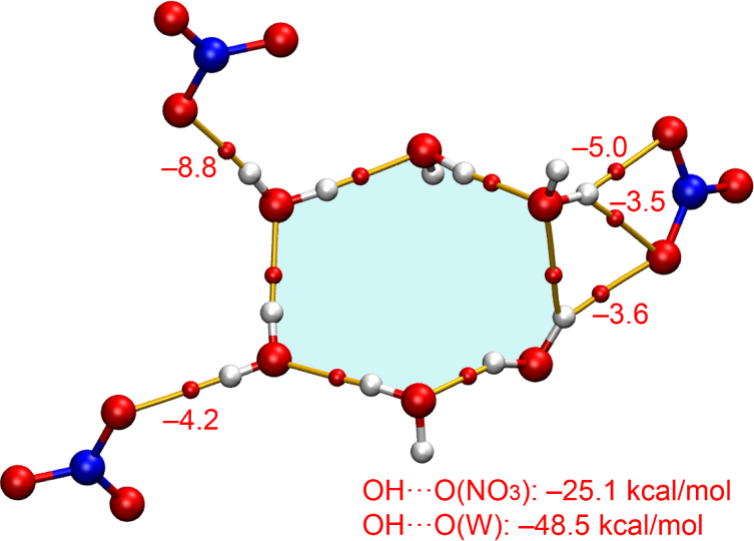
QTAIM analysis (BCPs as red spheres and bond paths as orange lines) of the hexameric water cluster in the presence of three nitrate anions. The energies of the OH···N H-bonds are indicated in red adjacent to the BCPs.

The total interaction energy of the OH···N hydrogen bonds with the [Ni­(**N** ^**3**^ **L**)_2_]^2+^ units is −20.5 kcal/mol, whereas the sum of the OH···O­(nitrate) interactions amounts to −25.1 kcal/mol, indicating that the latter contributes more significantly to the overall stability of the assembly. In any case, the stabilizing effect of the water cluster is considerably greater than that of the anion−π or CH···O (nitrate) interactions described in Figure 10, further emphasizing the critical role of the water cluster in maintaining the structural integrity of the system.

### Optical Study

To find out the optical bandgap of the respective complexes, we measured the UV–vis–NIR absorption spectra. Figure 13a,b represents UV–vis–NIR absorption spectra corresponding to complexes **1** and **2**, respectively. A prominent and similar absorption feature is seen in both complexes. We have determined the optical bandgap of the complexes with the help of the following Tauc relation

1 $$\left(\alpha h\nu \right)=A{\left(h\nu -E_{g}\right)}^{n}$$

**Figure 13:**
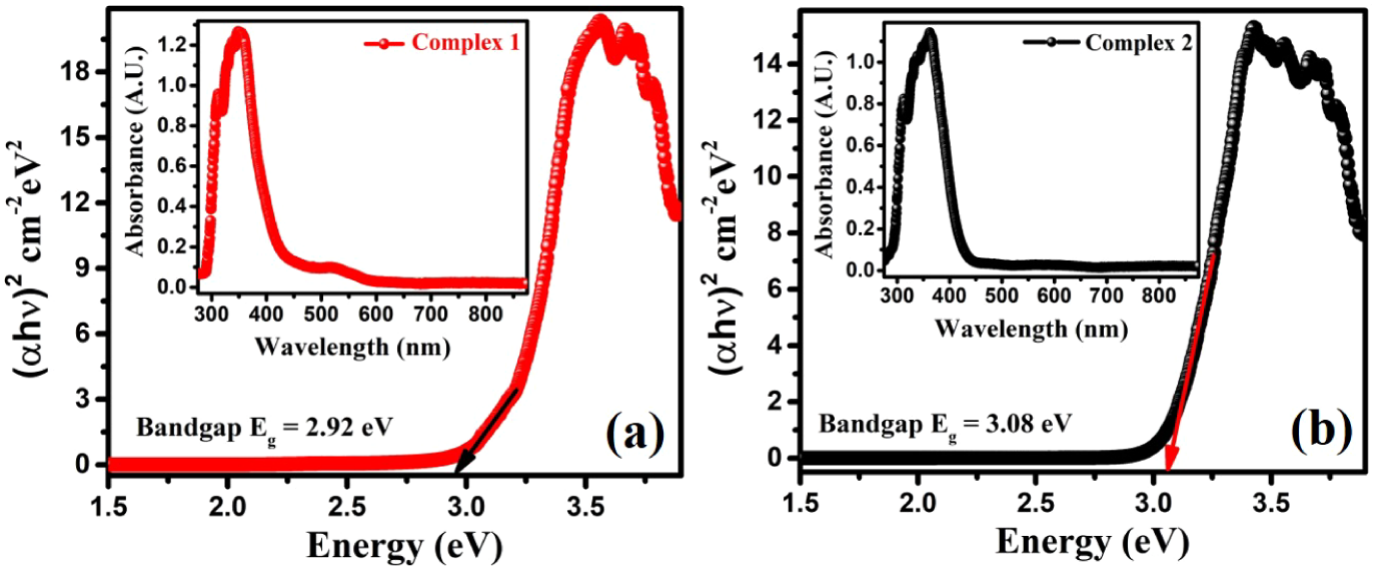
Tauc plots [(α*h*ν)^2^ vs *h*ν] to estimate the optical bandgaps of (a) complex **1** and (b) complex **2**. The insets show the corresponding UV–visible absorption spectra.

where α is the absorption coefficient, *hν* is the photon energy, *A* is a proportionality constant (which is independent of wavelength), *E*_g_ defines the optical bandgap of the complex, and for direct band-to-band transition, the coefficient *n* =1/2. According to eq 1, the absorption coefficient’s wavelength dependence must linearize in Tauc’s plot [(α*h*ν)^2^ vs *h*ν], and the material’s optical bandgap may be found at the location of the *x*-axis intercept for α = 0. For complexes **1** and **2**, the bandgaps (*E*_g_) were found to be 2.92 and 3.08 eV, respectively (see Figure 13). These results imply that, as these materials exhibit favorable electrical transport characteristics (such as conductivity and mobility), we should look into their potential for UV photodetection applications.

### Electrical Study

The current (*I*) vs voltage (*V*) characteristics of both devices were measured in the dark, at room temperature, and in the applied voltage range of −2 V to +2 V. The schematic illustration of the sandwiched Schottky device structure is shown in Figure 14a. The *I*–*V* characteristic curves provided by both devices in dark conditions are shown in Figure 14b, which illustrate the distinctive nonlinear and rectifying behavior of Schottky barrier diodes (SBDs). It is observed that, at ±2 V, device 1 has a greater (20) current rectification ratio (i.e., the ratio of the forward voltage’s current to the reverse voltage) than device 2 (9). This suggests that device 1 (complex **1**) will perform better as a rectifier device in comparison to device 2 (complex **2**). The conductivity measurement of the devices suggests that device 1 has a better ability to transport electrons than device 2. Through the examination of the linear segments of the *I*–*V* characteristic curves, room temperature conductivity (σ) was determined to be 3.73 ×10^–3^ S cm^–1^ and 7.2 × 10^–4^ S cm^–1^ for device 1 and device 2, respectively. We see that the room temperature conductivity, on an absolute scale, might not be high enough, but these values are much higher than the reported values for many Co­(II) or Ni­(II) complexes (Table S8), indicating the potential possibility of complexes **1** and **2** for optoelectronic applications.

**Figure 14:**
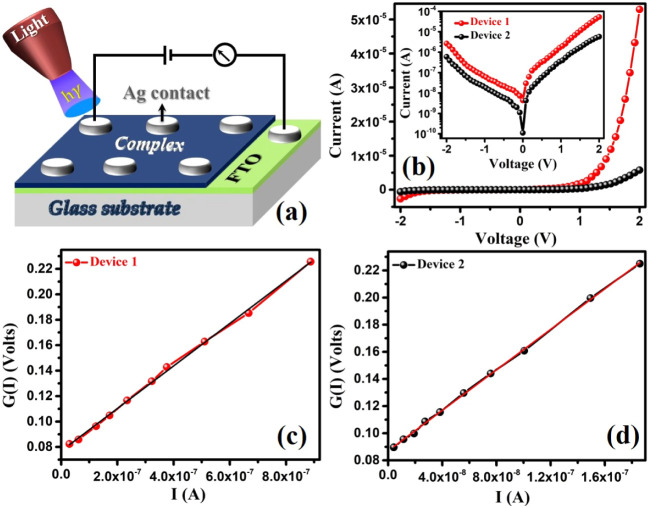
(a) Schematic presentation of the fabricated Schottky device (diode) structure. (b) *I*–*V* characteristic curves from Schottky devices 1 and 2 under dark conditions. The inset figure represents the current in logarithmic scale as a function of voltage. (c,d) Display *G*(*I*) vs *I* graphs under dark conditions, obtained from device 1 (complex **1**) and device 2 (complex **2**), respectively.

To fully understand these rectifying *I*–*V* curves and the primary charge transfer mechanisms, we must study them in detail. The devices’ rectifying *I*–*V* characteristics and nonlinear characteristics can be explained through the application of a Richardson–Schottky equation that takes into account thermionic emission and diffusion of carriers over barriers.^75,76^ Here, we can apply thermionic emission theory to study the present situation, as these complexes possess wide bandgap in nature. This theory states that the dark *I*–*V* characteristics of the Schottky devices can be represented by the equation,^76^

2 $$I=I_{0}\left[\exp\left(\frac{q_{e}V}{\eta k_{B}T}\right)-1\right]$$

where *I* and *I*_0_ are the forward and reverse saturation currents, respectively, *V* is the applied voltage, η is the ideality factor of the Schottky diode, *q*_e_ is the elementary charge, *K*_B_ is the Boltzmann constant, and *T* is the absolute temperature. Whereas the reverse saturation current (*I*_0_) can be written as^75,76^

3 $$I_{0}=AA^{*}T^{2}\exp\left(-\frac{q_{e}\Phi_{B}}{k_{B}T}\right)$$

where *A* defines the active device area (=3.14 × 10^–6^ m^2^), A* (= 132 A K^–2^ cm^–2^) is the Richardson constant, Φ_B_ is the Schottky barrier height (SBH), *K*_B_ is the Boltzmann constant, and *T* is the absolute temperature. Taking diode series resistance (*R*_s_) into account, the forward *I* vs *V* characteristics for can be expressed as^75,77^

4 $$I=I_{0}\left[\exp\left(\frac{q_{e}\left(V-IR_{s}\right)}{\eta k_{B}T}\right)\right]$$

where *IR*_s_ stands for the voltage drop across the series resistance *R*_s_. Cheung et al. have deduced eq 5 by combining the previous two equations (eqs 3 and 4).^77^ Equation 5 can be used to calculate the series resistance (*R*_s_) and ideality factor (η) of Schottky barrier devices based on the concerned materials (complex **1** and complex **2**).

5 $$G\left(I\right)=\frac{dV}{d\left(\ln I\right)}=\frac{\eta k_{B}T}{q_{e}}+IR_{s}$$

Now we can determine the ideality factor (η) from the intercept of *G*(*I*) vs *I* graph (straight line) along the *Y*-axis, and its slope will give the value of series resistance (*R*_s_). *G*(*I*) vs *I* graphs corresponding to device 1 and device 2 are displayed in Figure 14c,d, respectively. The ideality factor (η) for device 1 is found to be 2.95, whereas for device 2, it is 3.35. It is evident that the obtained η values for both devices are higher than the ideal case (η = 1). This implies that improper metal–semiconductor interfaces form in the SBDs, most likely as a result of defect or trap states present in the junction. Structural disorder in the materials is known to have an impact on the quality of the thin films and produce more cluttered structures on the surface, which leads to the formation of additional defect states at the metal–semiconductor interfaces. Because of these possibilities, the η values significantly differ from the ideal value of 1. Surface contamination, barrier tunneling, and other factors can also potentially be responsible for the Schottky diode’s nonideality.^78,79^ Simultaneously, the series resistance (*R*_s_) values for device 1 and device 2 are determined to be 167 ± 10 kΩ and 746 ± 15 kΩ, respectively. Therefore, device 2 (complex **2**) offers more than 4 times higher resistivity than device 1 (complex **1**), implying the formation of a comparatively better metal–semiconductor junction in device 1, and better charge transportation is expected.^80,81^ These findings further complement the conductivity (σ) results, where complex **1** showed about five times higher conductivity than complex **2**.

For accurate determination of the zero-bias Schottky barrier height, we have carried out temperature-dependent *I* vs *V* measurements in the temperature range of RT (298 K) to 363 K for both devices, as shown in Figure 15 a,b, respectively. Here, we mainly focus on the variation of reverse saturation current (see eq 3), particularly the current at zero voltage as a function of temperature. Equation 3 can be rewritten as follows:^82^

6 $$\ln\left(\frac{I_{0}}{T^{2}}\right)=\ln\left(AA^{*}\right)-\frac{q_{e}\Phi_{B}}{k_{B}T}$$

now by plotting the $\ln\left(\frac{I_{0}}{T^{2}}\right)$ vs $\frac{q_{e}}{k_{B}T}$ graph, we can calculate the zero-bias Schottky barrier height (Φ_B_) from the slope.^82^ The Arrhenius plots of $\ln\left(\frac{I_{0}}{T^{2}}\right)$ vs $\frac{q_{e}}{k_{B}T}$ for devices 1 and 2, shown in Figure 15c,d, display a linear temperature dependence in the range of 298 K–363 K. From Figure 15c,d, zero-voltage Schottky barrier heights for devices 1 and 2 are determined to be Φ_B_ = 0.37 eV and Φ_B_ = 0.49 eV, respectively. Therefore, device 1 offers a lower barrier height than device 2, which is in line with previous findings. It has to be emphasized that the obtained Φ_B_ values are quite precise because they are determined from the temperature dependence of the reverse saturation current, which does not require the exact value of the active Schottky contact area.^82^

**Figure 15:**
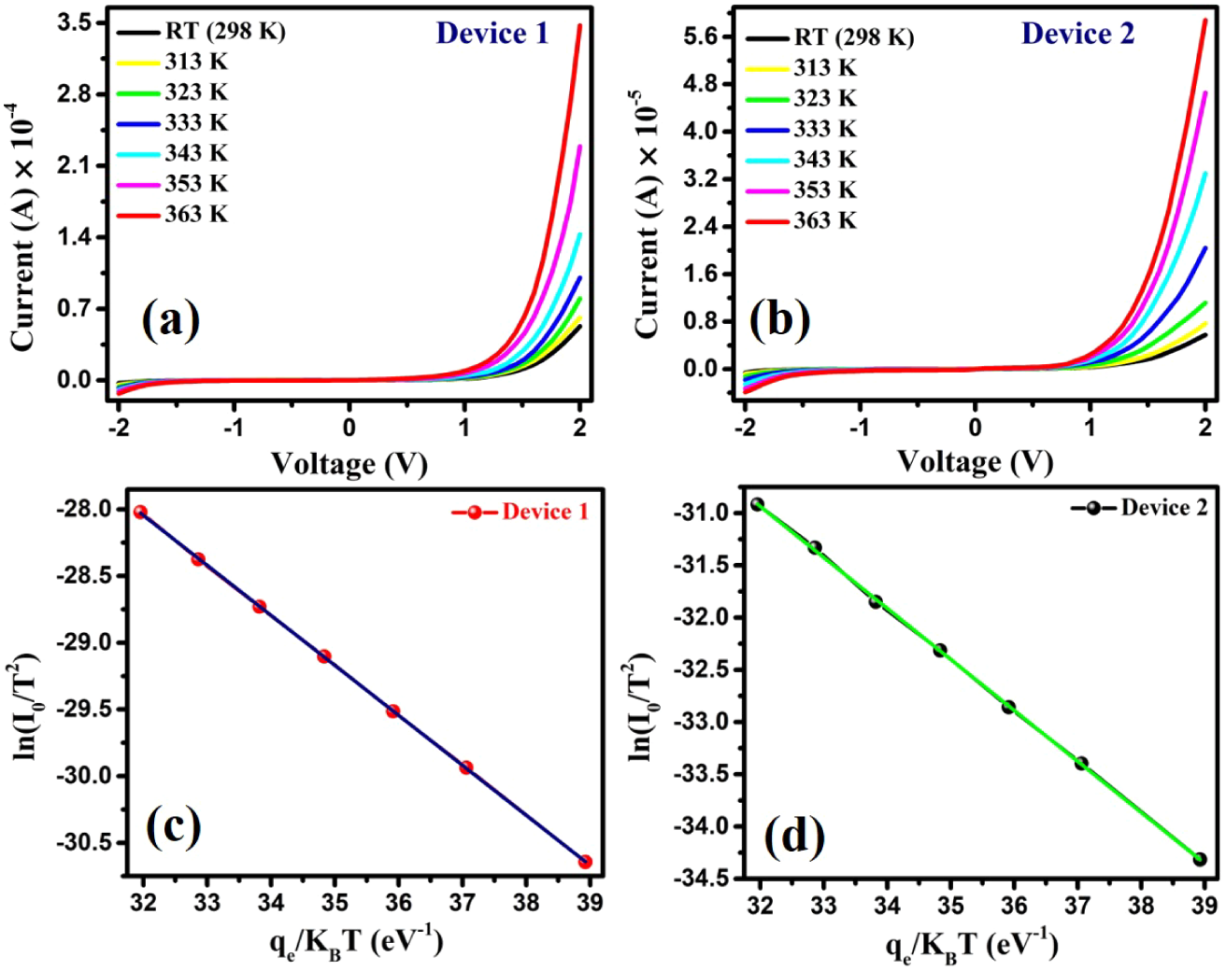
(a,b) Show temperature-dependent *I* vs *V* curves from devices 1 and 2, respectively. (c,d) Linear fitting to the Richardson plots (i.e., $\ln\left(\frac{I_{0}}{T^{2}}\right)$ vs $\frac{q_{e}}{k_{B}T})$ corresponding to the device 1 and device 2 has been employed to determine the zero-bias Schottky barrier height (Φ_B_).

To gain a detailed insight into the charge transport phenomena across the metal–semiconductor interface, we have plotted forward bias ln­(*I*) vs ln­(*V*) curves for both devices 1 and 2, as illustrated in Figure 16a,b, respectively. Figure 16a,b clearly show ln­(*I*) vs ln­(*V*) curves containing two distinct linear regions (designated as Region-I and Region-II) having different slopes, and therefore the charge transport mechanism is governed by the power law (*I* ∝ *V* ^m^).^83^ In Region–I, i.e., in the low-forward voltage region, both devices exhibit slopes ≈1 (*I* ∝ *V*), which is characteristic of Ohmic behavior; hence, this region is referred to as the Ohmic region.^83–85^ On the other hand, in Region–II (Figure 16a,b), slopes are close to the value 2 (i.e., *I* ∝ *V* ^2^), which implies that current conduction across the metal–semiconductor heterojunctions in both cases is dominated by a trap-charge-free, space-charge-limited current (SCLC) conduction mechanism.^83–85^ If the number of injected carriers exceeds that of background carriers, then the injected carriers spread out, leading to the formation of a space charge field. This space charge field controls the flow of space-charge-limited currents (SCLCs).^83^

**Figure 16:**
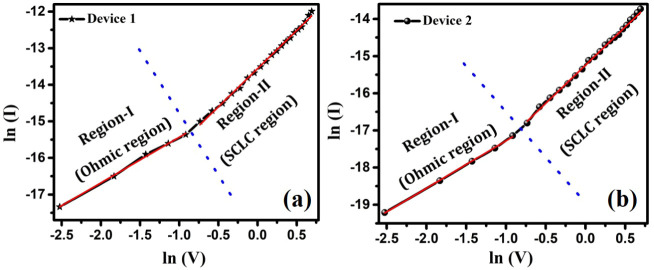
(a,b) Display forward-bias ln­(*I*) vs ln­(*V*) characteristic for device 1and device 2, respectively.

We also have determined the dielectric constants (ϵ_r_) of the concerned materials by measuring capacitance as a function of frequency, using the following eq 7:^84,85^

7 $$\epsilon_{r}=\frac{CD}{\epsilon_{0}A}$$

where *C* is the saturation capacitance, ϵ_0_ is the permittivity of free space, *D* is the thin film thickness (i.e., the vertical distance between two electrodes in the Schottky barrier diodes), and *A* is the active device area (the area of the contact, 3.14 × 10^–6^ m^2^). Figure S10a,b represents capacitance versus frequency plots for devices 1 and 2, respectively. It is observed that the capacitance values for both devices tend to saturate in the higher frequency regimes. From eq 7, the dielectric constant values corresponding to complex **1** (device 1) and complex **2** (device 2) are determined to be 0.12 and 0.83, respectively, which further complement the transport properties exhibited by the concerned materials. From our previous studies,^35,47^ we have established that the charge transport in these Schottky devices is mainly governed by the space charge limited current (SCLC) conduction mechanism. This behavior indicates the presence of traps or defects at the metal–semiconductor interfaces. Thus, the charge storage mechanism in both devices can be attributed to interfacial charge accumulation at these traps or defect sites. All the device properties related to both the Schottky barrier diodes (SBDs), determined from the electrical measurements performed in dark conditions at room temperature and with temperature variations, have been tabulated in Table S7.

In metal complexes, the bandgap is considered the easiest way to explain electrical conductivity. The interaction between the metal ions, d-orbitals, and the p-orbitals of the organic ligands results in the formation of extensive electronic networks. These networks enhance charge transport and contribute to the materials’ lower bandgaps, creating efficient conduction pathways that enable semiconducting behavior.^86^ The lower bandgap (responsible for higher conductivity) observed in complex **1** (2.92 eV) indicates comparatively higher electrical conductivity than that of complex **2** with higher bandgap (3.08 eV). Additionally, electrical conductivity depends not only on the bandgap value but also on the number of unpaired electrons that conduct electricity in the complexes via through-bond extended conjugation and through-space interactions involving weak interaction strategies.^35^ From a structural point of view, it is observed that the Co­(II) complex (complex 1) is a d^7^ system (t_2g_^5^e_g_^2^) with three unpaired electrons, whereas the Ni­(II) complex (complex **2**) contains only two unpaired electrons (d^8^ system, t_2g_^6^e_g_^2^). As both complexes exhibit almost the same supramolecular arrangements, the electrical conductivity depends mainly on the unpaired electrons present in the d orbital of the central metal ions. As complex **1** has a higher number of unpaired electrons, it demonstrates superior conductivity compared to complex **2** due to more efficient charge transport. This conclusion is supported by DFT calculations, which reveal a smaller bandgap for complex **1**.^87,88^ Therefore, the comparatively higher electrical conductivity as well as the better SBD made of complex **1** can be explained by the combined effects of its lower bandgap and greater number of unpaired electrons (present in complex **1**) than that of complex **2**.

In essence, we have investigated how the structural parameters, particularly the coordination geometry, metal–ligand bond lengths, and supramolecular interactions, influence the optical and electrical properties of the Co­(II) and Ni­(II) complexes. Regarding coordination geometry, both complexes exhibit distorted octahedral structures; however, the extent of distortion and ligand field environment differ slightly due to the nature of the central metal ion (Co vs Ni). This difference affects the d–d transitions and electronic distribution, which, in turn, influence their optical absorption and bandgap. Complex **1** (Co) shows a lower bandgap, making it more suitable for electronic applications. Shorter and slightly stronger Co–N bonds in complex **1** contribute to better overlap of metal d-orbitals with ligand orbitals, which enhances electronic delocalization, thereby improving the conductivity and reducing the Schottky barrier height in the device. Crystal packing and dimensionality, along with the presence of π···π stacking and anion···π interactions, lead to enhanced intermolecular electronic communication, especially in complex **1**. These interactions facilitate charge transport pathways, which are crucial for improving electrical performance. Additionally, the hydrogen-bonded (H_2_O)_6_ clusters contribute to crystal stability, indirectly supporting consistent electronic behavior.

### Theoretical DFT Study of Optical Properties

To assess the importance of d-electrons and validate the previous explanation, the crystal structure of compound **1** was modeled using DFT, maintaining the experimental crystal lattice. Compound **1** was chosen due to its slightly better electrical conductivity, as inferred from its marginally lower bandgap (by 0.1 eV) compared to compound **2**. To evaluate the agreement between the experimental data and theoretical predictions, we applied a standard band theory methodology. However, it is well known that DFT tends to underestimate semiconductor bandgap values.^89,90^ To mitigate this issue, we systematically incorporated a 1.4 eV scissor operator, which provided a more accurate representation of the electronic structure and enabled a meaningful comparison between theoretical simulations and experimental observations.

The band structure and total density of states (TDOS) diagram (Figure 17) confirm that the crystal exhibits semiconductor behavior. Both the valence and conduction bands are predominantly composed of p-type orbitals (green line), highlighting the significant contributions of the nitrate anions and aromatic rings. The maximum of the conduction band for the alpha electrons (centered around 2.5 eV, relative to the Fermi level *E*_f_) is at a lower energy compared to that of the beta electron band (centered around 2.9 eV). However, despite these differences, the bands remain p-electron dominated, indicating substantial spin density transfer from the metal center to the organic ligands. The three green bands at the lower part of the figure correspond to low-populated alpha electron bands.

**Figure 17:**
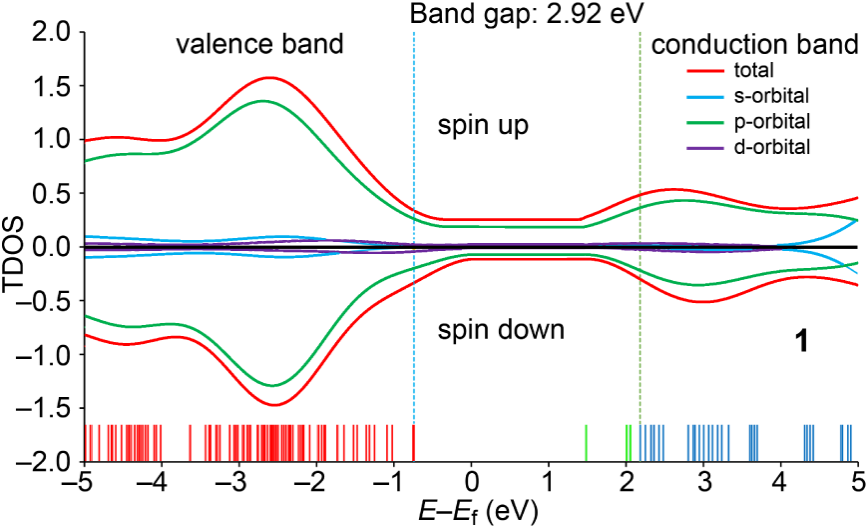
Total density of states and band structure of compound **1**. The red line corresponds to the sum of all orbitals, the blue line corresponds tothe s-orbitals, the red line corresponds to the p-orbitals, and the purple line corresponds to the d-orbitals.

The partial density of states (PDOS) plots were also computed to provide deeper insight into the electronic structure, as shown in Figure 18. The PDOS is presented separately for the ligand (top panel), the anions (middle panel), and the metal center (bottom panel). The analysis reveals that the highest valence band is significantly influenced by the p-orbitals of the anions, while the lower energy levels of the valence band are primarily dominated by the organic ligand. Additionally, the d-orbitals of the metal center contribute to the upper region of the valence band, though to a lesser extent.

**Figure 18:**
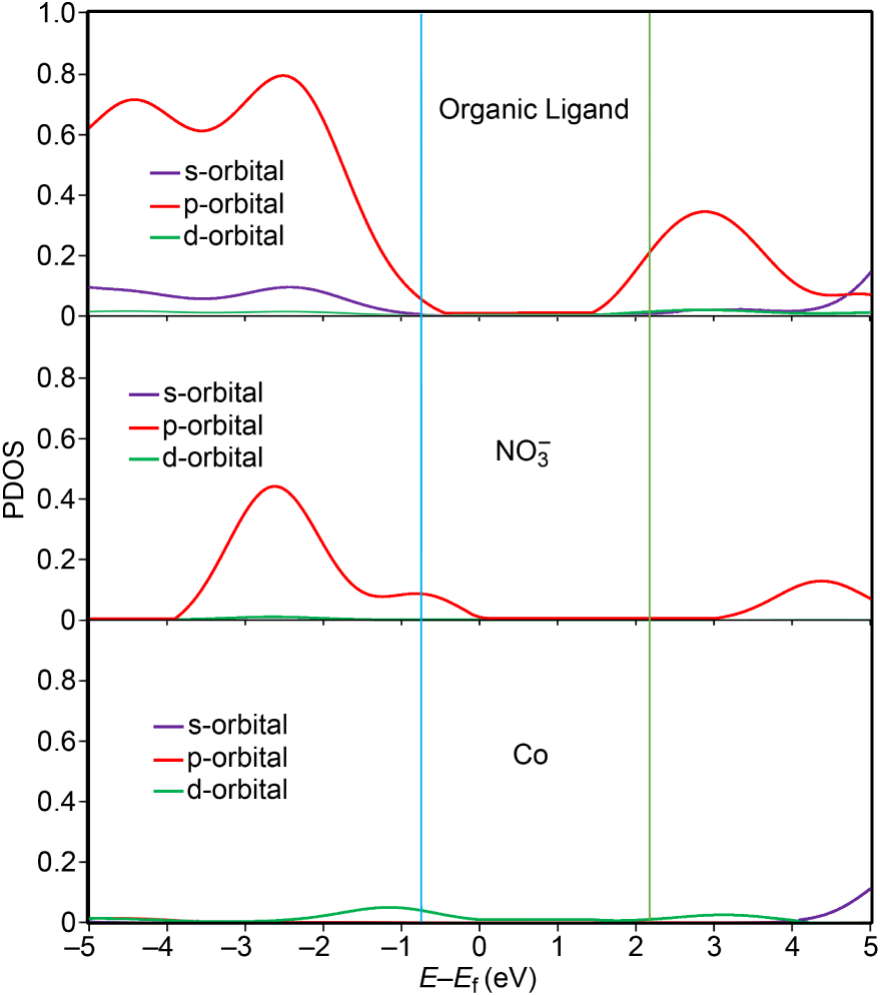
Calculated partial density of states for **1**. Top: organic ligands, middle: NO_3_^–^ anions, and bottom: Co metal. The lines illustrate the s-orbital (purple), p-orbital (red), and d-orbital (green) characteristics of the atoms within the compound.

In contrast, the lowest energy bands of the conduction level are exclusively attributed to the p-orbitals of the ligand, indicating that charge transport is primarily mediated through the organic framework. This suggests that the anion−π and CH···anion interactions play a key role in the semiconducting behavior of the system. Furthermore, the results reinforce that subtle differences in the bandgap can be attributed to the metal center and the number of unpaired electrons, as the d-orbitals of the metal center exhibit some influence on the upper valence band.

## Conclusions

This work is mainly focused on the successful synthesis and comprehensive physicochemical characterization of two novel transition metal complexes incorporating cobalt­(II) and nickel­(II) ions coordinated with a strategically designed tridentate **N** ^**3**^ **L** donor ligand. This work represents a synergistic integration of experimental methodologies—including spectroscopic and structural techniques—with advanced theoretical analyses like DFT, QTAIM, and NCI plot to provide deep insights into the role of metal ion variation.

The synergistic interplay of multiple noncovalent interactions (π···π, anion···π, and hydrogen bonding) collectively influences both the structural and electronic behaviors of the complexes. In particular, these interactions enhance charge delocalization, improve orbital overlap, and create efficient pathways for charge transport.

These interactions were found to be crucial in maintaining the structural integrity of the complexes and are likely to influence their optical properties, as suggested by the PDOS results. Electrical characterization of the fabricated Schottky diodes revealed that complex **1** exhibits superior rectification behavior and lower series resistance compared to complex **2**, making it a potentially more promising candidate for optoelectronic applications. Temperature-dependent *I–V* measurements further confirmed the lower Schottky barrier height of complex **1**, supporting its enhanced charge transport properties. We are not hesitant to say that although both complexes show relatively low electrical conductivity on an absolute scale, the complexes have much higher potential than those reported so far for Co­(II) or Ni­(II) complexes. Future studies could explore the tunability of these properties through modifications of the ligand or metal center, paving the way for the development of tailored materials with optimized performance for specific applications. Currently, syntheses of more appropriate ligands with π-conjugated systems and their various transition metal complexes for better charge transport properties and environmental stability, especially under humid conditions and substantial thermal strength that should not limit the long-term performance of advanced materials are underway.

## Supplementary Material











## Notes

Spectral data FTIR; crystal data table; hydrogen-bonding parameters; geometrical parameters for π···π, anion···π interactions; supramolecular assemblies; hexameric water cluster; optical characterization; device fabrication; device parameters (PDF)
checkCIF/PLATON report of complex **1** (PDF)
checkCIF/PLATON report of complex **2** (PDF)
Crystallographic data of complex **1** (CIF)
Crystallographic data of complex **2** (CIF)
