# Supplementary material for: Comparative Studies on the Structural, Optical, and Electrical Properties of Two [Co(II) and Ni(II)] Complexes: Insights through Theoretical Analysis
Source: J Phys Chem B. 2025 Jul 3;129(28):7389–405. doi: 10.1021/acs.jpcb.5c02577 (PMC12278210; doi:10.1021/acs.jpcb.5c02577)
Supplement: Supplementary file 1 [file jp5c02577_si_001.pdf]

SUPPORTING INFORMATIONS

Comparative Studies On Structural, Optical And Electrical Properties of Two [Co(II) And Ni(II)] Complexes: Insights Through Theoretical Analysis

Subhajit Saha[a], Samit Pramanik[b], Sudipta Pathak[c], Riya Sadhukhan[d], Arnab Ghosh[e], Dipak K. Goswami[d], Hon Man Lee*[,f], Rosa M. Gomila[g], Antonio Frontera*[,g] and Subrata Mukhopadhyay*[,a]

[a]Department of Chemistry, Jadavpur University, Kolkata 700032, India

[b]Department of Basic Science and Humanities, Alipurduar Government Engineering and Management College, Bakla 736206, India

[c]Department of Chemistry, Haldia Government College, Debhog, PurbaMedinipur, West Bengal, India

[d]Organic Electronics Laboratory, Department of Physics, Indian Institute of Technology Kharagpur, Kharagpur 721302, India

[e]Department of Physics and Natural Science Research Centre of Belda College under Vidyasagar University, Belda, West Bengal 721424, India

[f]Department of Chemistry, National Changhua University of Education, Changhua 500, Taiwan 500

[g]Departament de Química, Universitat de les Illes Balears, Crta. deValldemossa km 7.5, 07122 Palma de Mallorca (Baleares), Spain

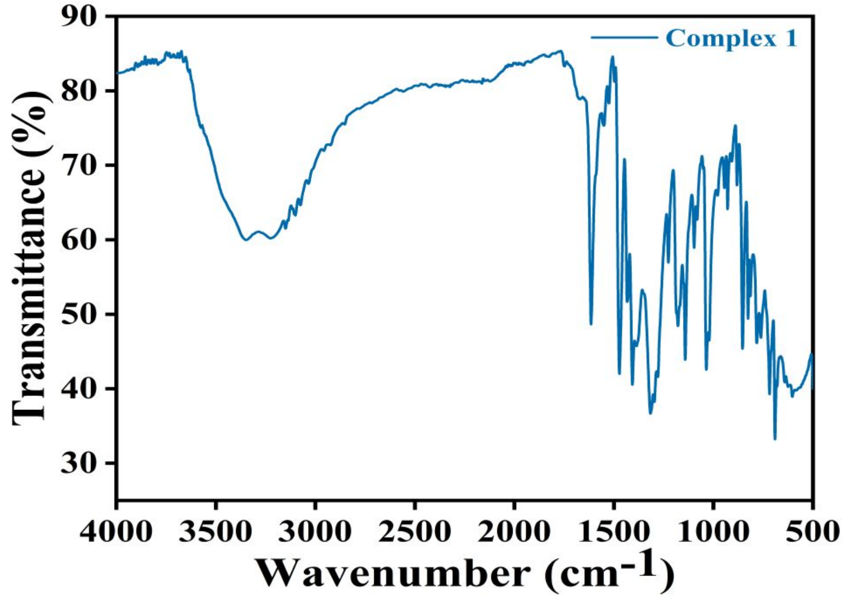

Figure S1. FT-IR spectrum of complex **1**

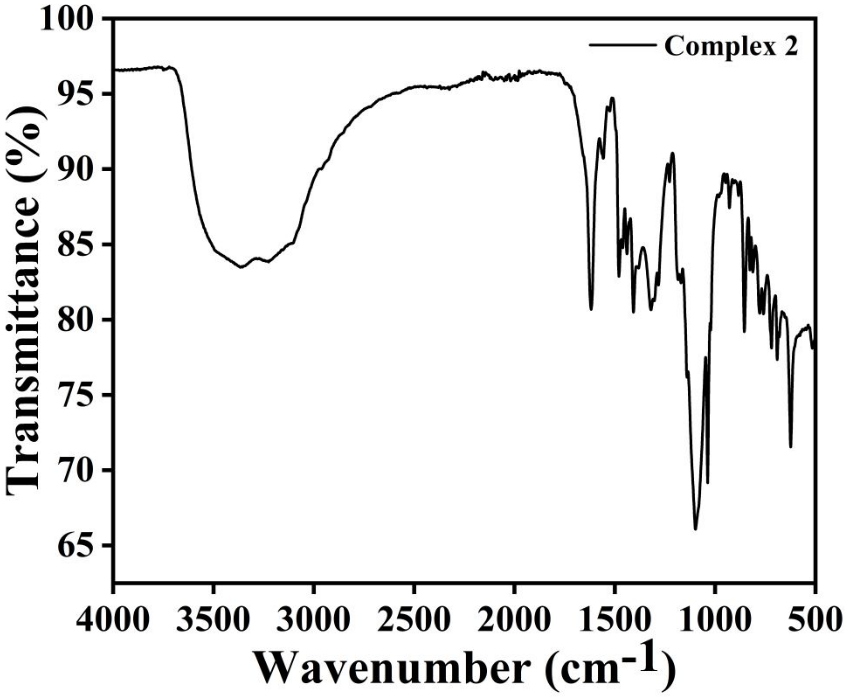

Figure S2. FT-IR spectrum of complex **2**

Table S1. Crystal Data and Structure Refinement Parameters for Complexes 1 and 2

Complex	1	2
Empirical formula	$C_{34}H_{38}N_{16}CoO_{12}$	$C_{34}H_{38}N_{16}NiO_{12}$
Formula Weight	921.73	921.51
Temperature (K)	100	99.98(10)
Wavelength (Å)	Mo Kα (λ = 0.71073)	Cu Kα (λ = 1.54184)
Crystal system	Triclinic	Triclinic
space group	P1̄	P1̄
a, b, c (Å)	9.96(3), 13.76(7), 14.67(5)	10.1109(2), 13.8068(3) 14.5968(3)
α, β, γ (°)	95.47(18), 98.52(12), 101.31(15)	94.968(2), 98.553(2), 102.076(2)
Volume ($Å^3$)	1932(13)	1955.79(7)
Z / Density (calc.) (g/cm^3)	2/1.584	2/1.565
Absorption coefficient (mm^{-1})	0.531	1.458
F(000)	954	956.0
Crystal size (mm^3)	0.18 × 0.15 × 0.12	0.19 × 0.16 × 0.14
θ range for data collection	1.941 to 25.999	6.168 to 149.68
Completeness to θ (%)	99.8%	93.9%
Absorption correction	Multi-scan	Multi-scan
Max. and min. transmission	0.7455 and 0.4720	1.000 and 0.871
Refinement method	Full-matrix least-squares on F^2	Full-matrix least-squares on F^2
Data/Restraints/parameters	7581/1/588	7556/0/619
Goodness-of-fit on F^2	1.0030	1.026
Final R in4dices [I > 2σ(I)]	R_1 = 0.0640, wR_2 = 0.1.552	R_1 = 0.0292, wR_2 = 0.0724
R indices (all data)	R_1 = 0.1008, wR_2 = 0.1727	R_1 = 0.0305, wR_2 = 0.0731
Largest diff. peak and hole (e.$Å^{-3}$)	0.699 and -0.725	0.46 and -0.40

Table S2. Selected Bond Distances [Å] for Complexes 1 and 2

	Bonds	Distance (Å)	Bonds	Distance (Å)
Complex **1**	Co1─ N2	1.868(10)	Co1─ N16	2.139(11)
	Co1─ N9	1.990(11)	Co1─ N22	2.099(11)
	Co1─ N10	1.913(10)	Co1─ N33	2.004(11)
Complex **2**	Ni1─ N2	1.9935(11	Ni1─N16	2.1123(12)
	Ni1─ N9	2.0953(12)	Ni1─N22	2.1031(12)
	Ni1─ N10	1.9939(11)	Ni1─N33	2.1058(12)

Table S3. Selected Bond Angles [°] for Complex 1 and Complex 2

	Bond angles	Value (°)	Bond angles	Value (°)
Complex **1**	N2─Co1─N9	82.0(2)	N9─ Co1─N33	161.4(2)
	N2─Co1─N10	177.9(2)	N10─ Co1─N16	78.2(2)
	N2─ Co1─N16	103.5(2)	N10─ Co1─N22	79.3(2)
	N2─ Co1─N22	99.2(2)	N10─ Co1─N33	101.7(2)
	N2─ Co1─N33	79.8(2)	N16─Co1─N22	157.1(2)
	N9─ Co1─N10	96.6(2)	N16─Co1─N33	86.7(2)
	N9─Co1─N16	93.8(2)	N22─Co1─N33	94.1(2)
	N9─Co1─N22	92.6(2)		
Complex **2**	N2─Ni1─N9	78.32(5)	N10─Ni1─N9	99.60(5)
	N2─Ni1─N10	177.56(5)	N10─Ni1─N16	78.10(4)
	N2─Ni1─N16	103.24(4)	N10─Ni1─N22	78.03(4)
	N2─Ni1─N22	100.74(4)	N10─Ni1─N33	104.34(5)
	N2─Ni1─N33	77.81(5)	N22─Ni1─N16	155.88(4)
	N9─Ni1─N16	94.16(4)	N22─Ni1─N33	95.17(4)
	N9─Ni1─N422	93.16(4)	N33─Ni1─N16	87.41(4)

	N9─Ni1─N33	155.79(5)

Table S4 Geometrical Parameters (Å, °) for the π-Stacking Interactions for the Title Complexes

Cg(i)···Cg(j)	Cg(i)···Cg(j)[Å]	α (°)	β (°)	γ (°)	Cg(i)–perp[Å]	Cg(j)–perp[Å]	Symmetry
**Complex 1**							
Cg(5)···Cg(5)	3.613(19)	0.02	1.55	1.55	3.611	3.611	2-x,-y,2-z
Cg(5)···Cg(10)	3.674(19)	11.91	16.98	15.93	3.532	3.513	1-x,-y,2-z
Cg(6)···Cg(6)	3.528(18)	0.03	9.05	9.05	3.484	3.484	-x,1-y,1-z
Cg(6)···Cg(12)	3.561(18)	12.08	16.30	20.88	3.327	3.418	1-x,1-y,1-z
Cg(10)···Cg(5)	3.674(19)	11.91	15.93	16.98	3.513	3.532	1-x,-y,2-z
Cg(12)···Cg(6)	3.561(18)	12.08	20.88	16.30	3.418	3.327	1-x,1-y,1-z
**Complex 2**							
Cg(5)···Cg(5)	3.5866(9)	0.03	4.1	4.1	3.5774	3.5774	2-x,-y,2-z
Cg(5)···Cg(10)	3.6474(8)	12.89	13.6	13.6	3.5444	3.5453	1-x,-y,2-z
Cg(6)···Cg(6)	3.5197(9)	0.00	9.3	9.3	3.4733	3.4733	-x,1-y,1-z
Cg(6)···Cg(12)	3.5230(8)	11.28	13.4	17.5	3.3601	3.4266	1-x,1-y,1-z
Cg(10)···Cg(5)	3.6474(8)	12.89	13.6	13.6	3.5453	3.5444	1-x,-y,2-z
Cg(12)···Cg(6)	3.5889(8)	11.28	17.5	13.4	3.4265	3.3600	1-x,1-y,1-z

Cg(i) and Cg(j) denotes centroid of ith and j^{th} ring respectively.

For Complex **1**: Cg(5) is the centroid of [N36/C35/C34/N38/C37] ring; Cg(6) is the centroid of [N46/C45/C44/N48/C47] ring; Cg(10) is the centroid of [N16/C17/C18/N19/C20/C21] ring; and Cg(12) is the centroid of [N30/C29/C28/N33/C32/C31] ring.

For Complex **2**: Cg(5) is the centroid of [N36/C35/C34/N38/C37] ring; Cg(6) is the centroid of [N46/C45/C44/N48/C47] ring; Cg(10) is the centroid of [N16/C17/C18/N19/C20/C21] ring and Cg(12) is the centroid of [N30/C29/C28/N33/C32/C31] ring.

Table S5. Geometrical Parameters (Å, °) for the Anion···π Interactions for the Title Complexes 1 and Complex2

Y─X(I)···Cg(J)	X···Cg [Å]	Y···Cg [Å]	Y─X···Cg (°)	Symmetry
**Complex 1**				
N(51)─O(50)···Cg(7)	3.059(16)	3.758(19)	114.7(3)	1-x,1-y,1-z
N(55)─O(57)···Cg(2)	3.361(17)	4.22(2)	126.7(3)	1+x,y,z
N(55)─O(57)···Cg(7)	3.373(17)	4.58(2)	165.9(3)	1+x,y,z
**Complex 2**				
N(51)─O(50)···Cg(7)	3.0049(13)	3.7687(13)	118.15(9)	1-x,1-y,1-z
N(55)─O(57)···Cg(2)	3.4085(13)	4.3075(15)	129.63(10)	x,y,z
N(55)─O(57)···Cg(7)	3.3977(13)	4.6180(14)	167.76(10)	x,y,z

Cg(j) denotes centroid of jth ring of the title complexes. For Complex **1**: Cg(2) is the centroid of [Co1/N2/C7/C8/N9] ring; and Cg(7) is the centroid of [N2/C3/C4/C5/C6/C7]] ring. For Complex **2**: Cg(2) is the centroid of [Ni1/N2/C7/C8/N9] ring, Cg(3) is the centroid of [Ni1/N10/C11/C27/N22] ring; and Cg(7) is the centroid of [N2/C3/C4/C5/C6/C7] ring.

Table S6. Geometrical Parameters for the Hydrogen Bonds of Complexes 1 and 2

D─H···A	D─H [Å]	H···A	D···A [Å]	D─H···A [°]	Symmetry
**Complex 1**					
O58─H58A···N46	0.8700	1.9200	2.788(15)	175.00	-
O58─H58B···O50	0.8700	2.1200	2.926(15)	154.00	-
O58─H58B···O52	0.8700	2.2200	2.968(16)	145.00	-
O59─H59B···O63	0.8700	1.9200	2.783(15)	170.00	-
O60─H60B···O58	0.8700	1.9100	2.723(15)	173.00	-

O61─H61A···O59	0.8700	1.9100	2.723(15)	155.00	-
O61─H61B···O52	0.8700	2.1700	2.917(15)	143.00	-
O61─H61B···O58	0.8700	2.4600	3.151 (17)	137.00	-
O62─H62A···O60	0.8700	1.8600	2.729(15)	173.00	-
O62─H62B···O54	0.8400	1.9300	2.746(15)	163.00	-1+x,y,z
O63─H63A···O62	0.8700	1.8000	2.656(14)	166.00	-
C17─H17···O57	0.9500	2.4700	3.184(17)	131.00	-1+x,y,z
C18─H18···O62	0.9500	2.5900	3.323(18)	134.00	-
C20─H20···O61	0.9500	2.3200	3.356(17)	168.00	1-x,1-y,2-z
C23─H23···O50	0.9500	2.4000	3.314(18)	161.00	1-x,1-y,1-z
C26─H26···O53	0.9500	2.3900	3.202(17)	143.00	x,-1+y,z
C29─H29···O50	0.9500	2.3100	3.243(17)	169.00	-
C34─H34···O54	0.9500	2.5400	3.344(18)	143.00	2-x,-y,2-z
C39─H39A···O52	0.9800	2.4900	3.312(18)	141.00	x,-1+y,z
C40─H40···O61	0.9500	2.3600	3.219(17)	149.00	x,-1+y,z
C41─H41···O59	0.9500	2.6000	3.383(18)	140.00	x,-1+y,z
C44─H44···O57	0.9500	2.5500	3.172(17)	124.00	1-x,1-y,1-z
C49─H49B···O53	0.9800	2.3400	3.302(18)	166.00	1-x,1-y,1-z
**Complex 2**					
O58─H58A···N46	0.93(2)	1.87(2)	2.7952(17)	179(2)	-
O58─H58B···O50	0.98(3)	2.02(3)	2.9525(18)	159(2)	-
O58─H58B··· O52	0.98(3)	2.16(3)	2.9678(18)	139(2)	-
O59─H59B···O63	0.90(3)	1.88(3)	2.770(2)	172(2)	-
O60─H60B···O58	0.98(3)	1.79(3)	2.7628(17)	172(3)	-
O61─H61A···O59	1.04(3)	1.77(3)	2.7428(19)	155(2)	-
O61─H61B···O52	0.98(3)	2.08(3)	2.9179(17)	142(2)	-
O61─H61B···O58	0.98(3)	2.41(3)	3.1731(17)	135(2)	-
O62─H62A···O60	0.91(2)	1.86(2)	2.751(2)	164(2)	-
O62─H62B···O54	0.92(3)	1.85(3)	2.7502(18)	169(3)	-
O63─H63A···O62	0.82(3)	1.87(3)	2.690(2)	175(3)	-
C17─H17···O57	0.9500	2.5100	3.2221(19)	132.00	-
C18─H18···O62	0.9500	2.5500	3.307(2)	137.00	-
C20─H20···O61	0.9500	2.3000	3.2319(19)	169.00	1-x,1-y,2-z
C23─H23···O50	0.9500	2.3700	3.2851(19)	162.00	1-x,1-y,1-z
C26─H26···O53	0.9500	2.4000	3.2157(19)	144.00	x,-1+y,z
C29─H29···O50	0.9500	2.2900	3.2236(19)	167.00	-
C34─H34···O54	0.9500	2.5100	3.3330(19)	144.00	1-x,-y,2-z
C39─H39A···O52	0.9800	2.4900	3.339(2)	144.00	x,-1+y,z
C40─H40···O61	0.9500	2.3400	3.2325(19)	156.00	x,-1+y,z
C44─H44···O57	0.9500	2.5600	3.1900(19)	124.00	-x,-y,1-z
C49─H49B···O53	0.9800	2.3700	3.342(2)	170.00	1-x,1-y,1-z

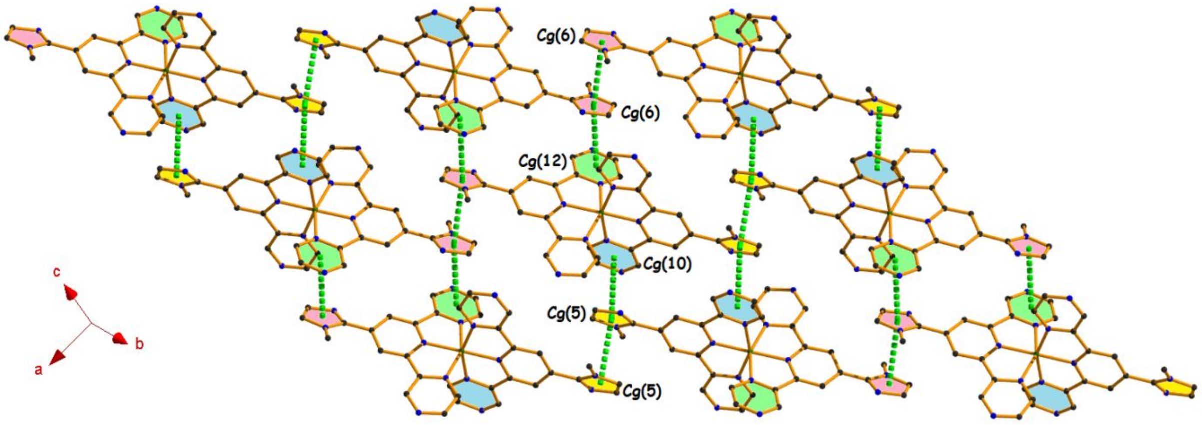

Figure S3. Perspective view of 2-D architecture through π•••π interactions in complex **2** (hydrogen atoms have been omitted for clarity).

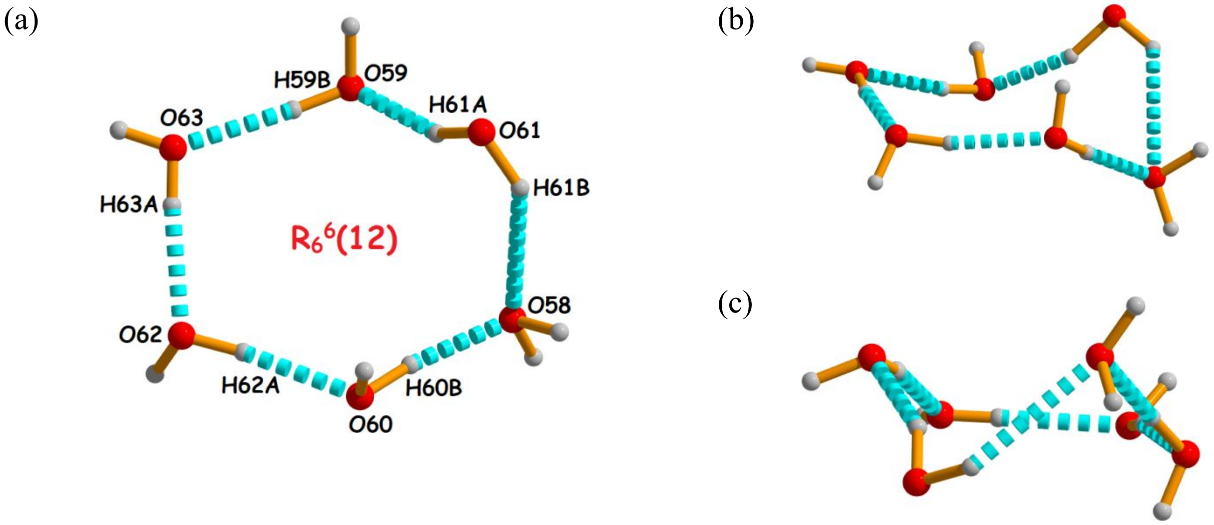

Figure S4. Perspective view of the hexameric water cluster with atom numbering scheme formed in complex 2 and the graphical representations of the water cluster as (a) ring like, (b) chair like and (c) twist-boat like.

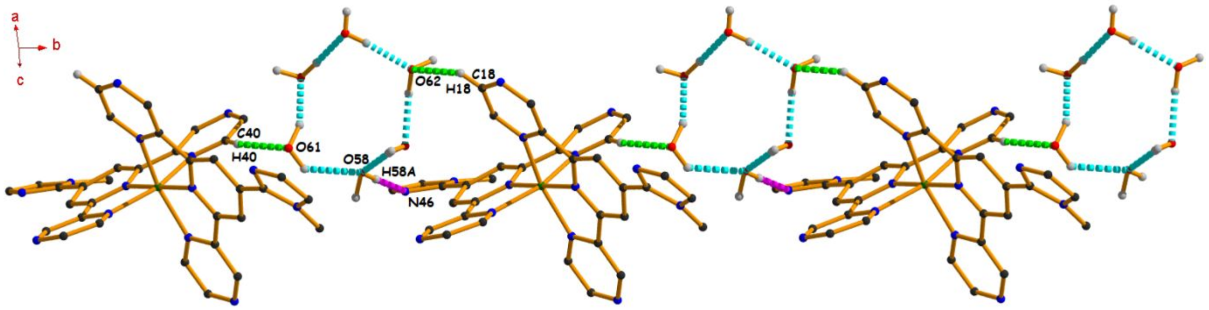

Figure S5. 1-D polymeric chain formation combining C─H•••O(shown by green dotted lines), O─H•••O(shown by cyan dotted lines) and O─H•••N(shown by pink dotted lines) hydrogen bonding interactions in complex **2** (other hydrogen atoms have been omitted for clarity).

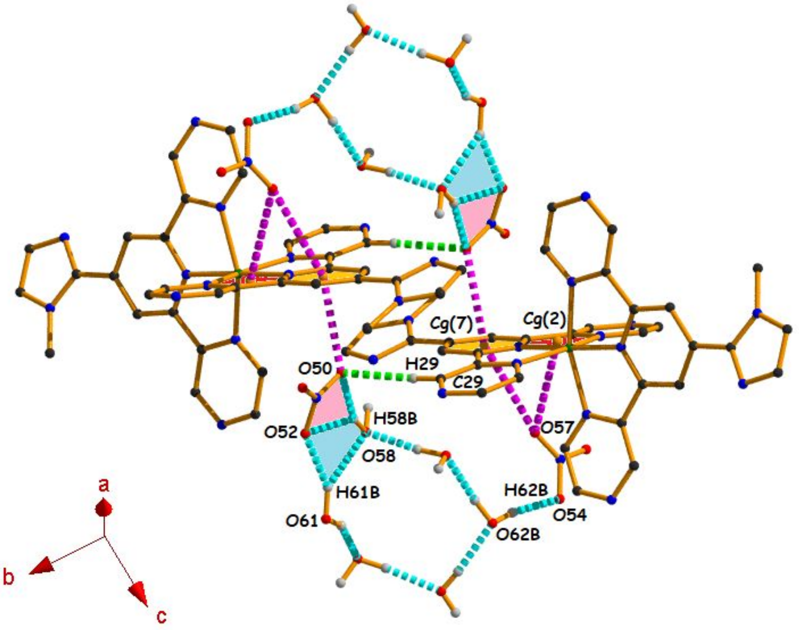

Figure S6. Supramolecular association involving anion•••π(shown by pink dotted lines) and hydrogen bonding interactions in complex **2** (other hydrogen atoms have been omitted for clarity).

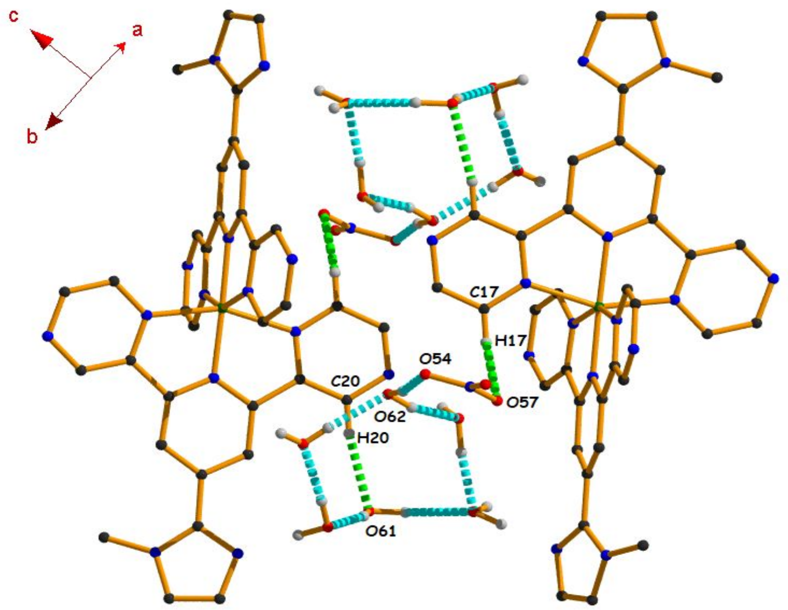

Figure S7. View of dimeric assembly formed by C─H•••O and O─H•••O hydrogen bonding interactions in complex **2** (other hydrogen atoms have been omitted for clarity).

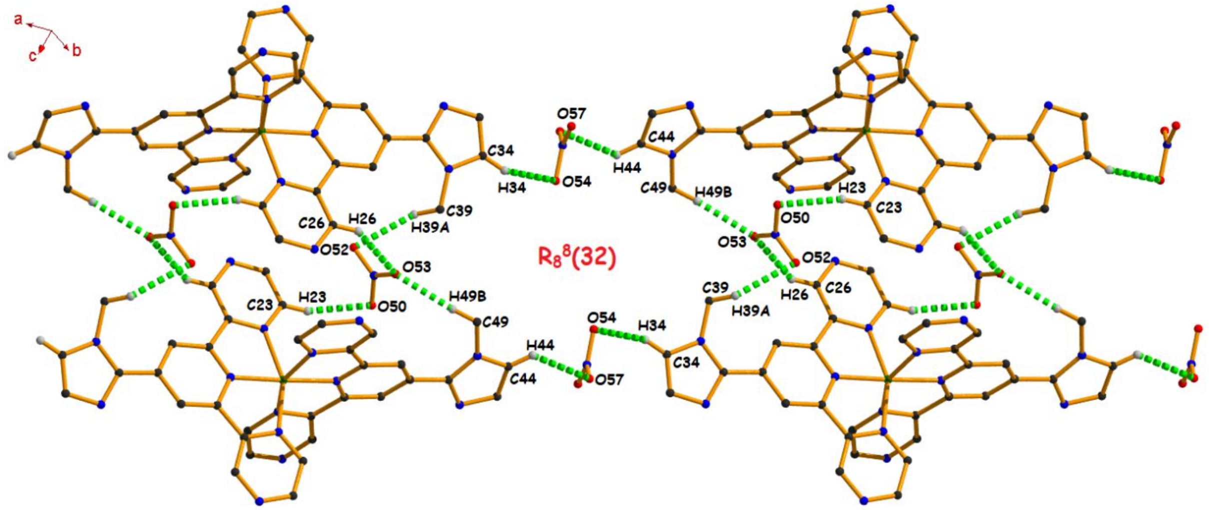

Figure S8. Formation of 1-D tape like architecture through C─H•••O hydrogen bonding interactions in complex **2** (other hydrogen atoms have been omitted for clarity).

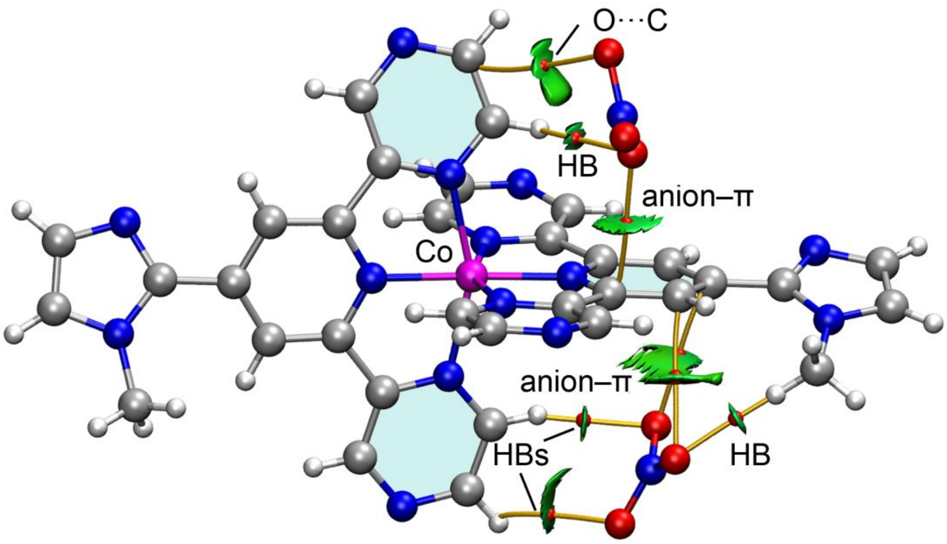

Figure S9. Combined QTAIM (BCPs as red spheres and bond paths as orange lines) and NCIPlot (RDG = 0.5, $\rho_{cut\text{-}off}$ = 0.04 a.u., color scale ±0.035 a.u.) analyses of the trimeric assembly of compound **3**. Only intermolecular interactions are indicated.

Fabrication of thin films and FTO/complex/Ag Schottky devices:

Good quality as-synthesized materials are the prerequisites for their utilization in the efficient electronic/optoelectronic devices. A uniform thin film is crucial for the construction of effective electrical/optoelectronic devices because it links the properties of macro systems and molecular systems. To fabricate devices, first, we prepared homogeneous solutions of complex **1** and **2** of concentration 5 mg/ml in N, N-dimethylformamide (DMF) solvent, through ultrasonication for 15min.FTO-coated glass substrates (sheet resistance ~9 Ω/cm^2, FTO thickness: 150 nm) were used for the fabrication of Schottky devices of surface area 1.5 × 1.5 cm^2. Substrates were sequentially cleaned in acetone, 2-propanol (IPA), and deionized water for 15 minutes in each solution under vigorous ultrasonication. A hot plate set at roughly 100 °C was then used for drying. Subsequently, a few drops of the prepared solutions were spin-casted onto the cleaned FTO/glass substrates at speeds of 700 rpm and 1200 rpm, respectively, for a minute each. The

films were then dried for 30 minutes at a temperature of around 70 °C on a hot plate to achieve a thickness of approximately 1 ± 0.1 μm. The sandwiched Schottky device fabrications were completed by taking Ag contacts, one on the FTO surface (bottom contact) and the other son the spin-coated thin film (top contacts) using a 2 mm diameter circular mask (to get an effective device area $\approx 3.14 \times 10^{-6}$ m^2), using a thermal evaporator at a base pressure of5×10^{-6} mbar. Atomic Force Microscopy (AFM, Asylum Research) was used to measure the thickness of the spin-coated films. Henceforth, Schottky barrier devices (SBD), made with complex 1 and complex 2 will be referred to as device **1** and device **2** respectively.

X-ray crystallographic analysis

Single-crystal X-ray data were obtained using using a Bruker SMART APEX II CCD area detector equipped with a graphite monochromated Mo Kα radiation (λ = 0.71073 Å) source in the φ and ω scan modes and a Rigaku XtaLabSynerg DW X-ray diffractometer equipped with a MicroMax-007 HF microfocus rotating anode that generated CuKα radiation (λ = 1.54184 Å) and a HyPix-Arc 150° curved hybrid photon counting X-ray detector at 100 K for complex 1 and complex 2 respectively. The data acquisition and reduction were carried out using the CrysAlisPro 1.171.42.51a software.[S1] Empirical absorption correction was performed using spherical harmonics implemented in SCALE3 ABSPACK. The structures of both the complexes were solved by conventional direct methods and refined by full matrix least-squares methods using F^2 data. SHELXT and SHELXL 2018/3 programs were used for the solution and refinement of the structure of all the complexes, respectively.[S2,S3] Final calculations were performed using Olex2 1.5.[S4] CCDC 2430019 (for complex **1**) and 2430020 (for complex **2**) contain the supplementary crystallographic data for this work.

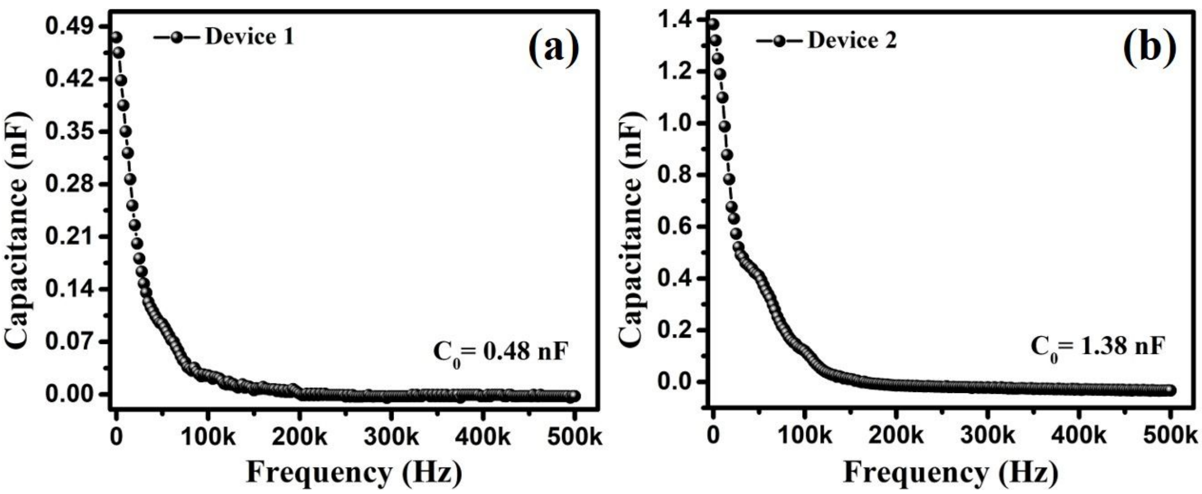

Figure S10: (a-b) Represent capacitance as a function of frequency plots for device 1 and device 2 respectively.

Table S7: Schottky barrier diodes (device 1and device 2) parameters under dark conditions

**Device**	**Rectification ratio (± 2 V)**	**conductivity σ (S cm^{-1})**	**Ideality factor (η)**	**Series Resistance R_s (kΩ)**	**Barrier height ϕ_B(eV)**	**Dielectric constant (ϵ_r)**
device 1	20	3.73×10^{-3}	2.95	167 ± 15	0.37	1.2×10^{-1}
device 2	9	7.2×10^{-4}	3.35	746 ± 15	0.49	8.3×10^{-1}

(Performed at room temperature and with temperature variation)

(S1)

Table S8. Some representative conductivity values of Co(II) and Ni(II) complexes with a comparison of the present work

Sl. No.	Metal complexes	Conductivity in Sm^{-1}	References

1.	$C_{30}H_{36}Co_2N_4O_9S,0.33H_2O$	2.04×10^{-5}	1
2.	$C_{34}H_{44}Co_2N_4O_9S_2$	1.54×10^{-3}	
3.	$C_{26}H_{22}CoN_2O_6$	2.61×10^{-5}	2
4.	$C_{26}H_{20}Br_2CoN_2O_7$	2.52×10^{-6}	
5.	$C_{34}H_{40}N_8O_{12}Co$	5.76×10^{-4}	3
6.	$C_{34}H_{36}Br_4N_8O_{12}Co$	1.33×10^{-3}	
7.	$C_{48}H_{38}CoN_2O_6$	7.66×10^{-5}	4
8.	$C_{38}H_{50}Co_3N_{16}O_5.CH_3OH$	1.05×10^{-6}	5
9.	$C_{46}H_{65.33}Co_3N_4O_{14.67}$	5.858×10^{-4}	6
10.	$C_{58.04}H_{69.59}Cl_{1.59}Co_3N_4O_{16.78}S_{1.59}$	6.804×10^{-5}	
11.	$C_{62.50}H_{77.50}Cl_{1.50}Co_3N_4O_{16.50}S_{1.50}$	9.358×10^{-5}	
12.	$C_{28}H_{32}Co_2N_9O_8$	2.04×10^{-5}	7
13.	$C_{30}H_{36}Co_2N_9O_8$	2.58×10^{-4}	
14.	$C_{32}H_{22}N_8NiClO_9$	2.4×10^{-4}	8
15.	$C_{36}H_{28}N_{12}NiOF_{24}P_4$	6×10^{-4}	
16.	$C_{17}H_{11}N_5NiS_2$	7.0×10^{-5}	9
17.	$C_{18}H_{13}N_5NiS_2$	2.0×10^{-5}	
18.	$C_{32}H_{28}N_4NiCl_2O$	9.2×10^{-12}	10
19.	$C_{20}H_{18}N_4NiCl_2$	8.1×10^{-12}	
20.	$C_{38}H_{26}N_6NiCl_2$	2.6×10^{-7}	
21.	$C_{28}H_{22}N_6NiCl_2$	4.3×10^{-7}	
22.	$C_{26}H_{18}N_6NiCl_2$	1.1×10^{-7}	
23.	$C_{22}H_{22}N_4O_9Ni$	1.45×10^{-5}	11
24.	$C_{32}H_{22}N_{16}NiO_6$	5.52×10^{-5}	12
25.	$C_{32}H_{22}Cl_2N_{14}NiO_8$	1.66×10^{-4}	
26.	$Co(C_7H_8N_2O)Cl_2$	5.1×10^{-8}	13
27.	$Ni(C_7H_8N_2O)Cl_2$	3.6×10^{-8}	
29.	$Co(aminopyrazine)_2(CNS)_2$	3.2×10^{-9}	14
30.	$Ni(aminopyrazine)_2(CNS)_2$	6.4×10^{-7}	14
31.	$Co(aminopyrazine)_2Cl_2$	2.2×10^{-5}	

32.	Ni(aminopyrazine)$_2$Cl$_2$	8.7×10^{-7}	
33.	Co(schiefbase)(NO$_3$)(H$_2$O)	2.54×10^{-5}	15
34.	Ni(schiefbase)(NO$_3$)(H$_2$O)	13.7×10^{-5}	
35.	$C_{34}H_{38}N_{16}CoO_{12}$	3.73 × 10^{-1}	This Work
36.	$C_{34}H_{38}N_{16}NiO_{12}$	7.2 × 10^{-2}	

Cartesian coordinates

Anion-π

```
 8    -3.299490   -2.945191    0.441620
 8    -2.601568   -4.679279   -0.657540
 8    -3.600036   -3.068517   -1.708803
 7    -3.178172   -3.558410   -0.655433
28     0.478130    0.042675    0.311236
 7    -1.513330    0.053520    0.402663
 7    -0.039737    0.015222   -1.718802
 7     2.464511   -0.013558    0.148615
 7     0.973754    2.096022    0.315070
 7     1.993805    4.663706    0.637403
 7     0.861332   -2.024561    0.361236
 7     1.687985   -4.687774    0.333399
 7    -0.659191    0.746628    5.000259
 7     0.129047    0.221177    2.380259
 7     7.399499    0.981331    0.189890
 7     7.486100   -1.081587   -0.633457
 7    -1.050656    0.110529   -4.312310
 7    -6.378249    0.389249    1.700111
 7    -6.614874   -0.499445   -0.323327
 6    -2.130340    0.082406    1.594348
 6    -3.510705    0.027327    1.688524
 1    -3.936697    0.019980    2.537558
 6    -4.271010   -0.017343    0.508082
 6    -3.607136    0.009013   -0.718368
 1    -4.097640    0.017596   -1.531865
 6    -2.220231    0.022535   -0.732616
 6    -1.373545    0.029154   -1.946245
 6     3.089838   -1.195633    0.100992
 6     4.469367   -1.278215   -0.011644
 1     4.903054   -2.123399   -0.019757
 6     5.213759   -0.095502   -0.113491
 6     4.534284    1.132304   -0.077036
 1     5.011474    1.950139   -0.154259
 6     3.159094    1.132380    0.072940
 6     0.167880    3.133705    0.547800
 1    -0.770918    2.996908    0.596014
 6     0.685447    4.413360    0.721023
 1     0.089935    5.130589    0.904089
 6     2.796166    3.624425    0.376321
 1     3.730258    3.773665    0.289048
 6     2.302990    2.333814    0.228983
 6    -0.015583   -3.022506    0.488698
 1    -0.938944   -2.824810    0.593112
 6     0.404189   -4.346013    0.470431
 1    -0.245629   -5.033501    0.558664
 6     2.564382   -3.681948    0.212418
```

1	3.487428	-3.885031	0.116893
6	2.165232	-2.348392	0.222261
6	-1.177869	0.234355	2.722676
6	-1.552042	0.475594	4.040283
1	-2.474231	0.447081	4.267100
6	0.626006	0.750588	4.635182
1	1.287351	0.949435	5.287484
6	1.027879	0.474158	3.336408
1	1.953734	0.464559	3.124132
6	8.774011	-0.639788	-0.504606
1	9.563044	-1.120184	-0.726193
6	8.706351	0.620854	0.002828
1	9.456255	1.170421	0.198458
6	6.676876	-0.070767	-0.196373
6	7.127752	-2.375687	-1.219694
1	6.343112	-2.266873	-1.796810
1	7.879212	-2.713293	-1.750462
1	6.921086	-3.012616	-0.504125
6	0.770263	0.035451	-2.778477
1	1.711087	0.013292	-2.648769
6	0.258149	0.088854	-4.069511
1	0.861892	0.110219	-4.802629
6	-1.854266	0.068267	-3.246616
1	-2.794063	0.064934	-3.384882
6	-7.870938	-0.325261	0.192530
1	-8.693155	-0.542119	-0.231195
6	-7.713853	0.218016	1.428072
1	-8.423406	0.445885	2.017260
6	-5.735088	-0.050622	0.620078
6	-6.359614	-1.186632	-1.594621
1	-6.214879	-0.523320	-2.301324
1	-5.562300	-1.749895	-1.507191
1	-7.130934	-1.745780	-1.824415
8	-2.001949	5.080624	-1.421384
8	-2.305695	3.823456	-3.155472
8	-2.558271	2.998462	-1.164372
7	-2.295235	3.942420	-1.925918

Water cluster with two Ni(II) complexes:

28	5.250498	-0.131227	-0.066486
7	4.920053	-2.097136	-0.054046
7	6.174301	-0.705277	1.724337
7	5.653804	1.821231	-0.037812
7	3.537904	0.653260	0.889282
7	1.300010	2.039403	1.788344
7	7.071805	-0.046712	-1.114652
7	9.508579	0.388831	-2.398798
7	2.236582	-1.021982	-3.794482
7	4.056410	-0.392092	-1.781164
7	5.458880	6.846933	0.184530
7	7.653358	6.613777	-0.082257
7	7.192934	-1.765505	4.089905
7	3.330254	-6.824891	-0.818504
7	5.036492	-7.243795	0.542785
6	4.238360	-2.669516	-1.058825
6	4.048773	-4.040293	-1.107170
1	3.589240	-4.438597	-1.836940
6	4.546890	-4.832457	-0.059382
6	5.203876	-4.206794	1.000233
1	5.517688	-4.713975	1.739648
6	5.392051	-2.833152	0.958327
6	6.085395	-2.029269	1.989112
6	6.788280	2.269211	-0.588161
6	7.105843	3.618871	-0.589396
1	7.902704	3.926638	-1.005064
6	6.233139	4.521765	0.032273

6	5.056901	4.026498	0.616697
1	4.451391	4.613570	1.054207
6	4.793371	2.670349	0.545479
6	2.414866	0.011310	1.216216
1	2.379544	-0.935694	1.149724
6	1.294757	0.711595	1.652820
1	0.502489	0.230274	1.860887
6	2.437657	2.676821	1.485710
1	2.480106	3.619861	1.592591
6	3.558059	1.999924	1.020076
6	7.753568	-1.053569	-1.664768
1	7.403623	-1.935748	-1.621407
6	8.967948	-0.828360	-2.299413
1	9.431028	-1.567088	-2.676698
6	8.816740	1.396558	-1.850561
1	9.167122	2.277837	-1.904302
6	7.600624	1.192878	-1.204650
6	3.699042	-1.673315	-2.018021
6	2.806872	-1.971413	-3.042453
1	2.594546	-2.881343	-3.214603
6	2.586883	0.239562	-3.528738
1	2.194327	0.940920	-4.035074
6	3.504394	0.560560	-2.538857
1	3.743369	1.469123	-2.397780
6	7.388004	7.954774	-0.040859
1	8.020389	8.660242	-0.110560
6	6.042968	8.082878	0.119143
1	5.577371	8.909020	0.177101
6	6.455143	5.970383	0.052580
6	9.005873	6.057246	-0.170344
1	9.078651	5.280166	0.422459
1	9.657318	6.737189	0.101100
1	9.186362	5.781973	-1.093424
6	6.777055	0.068768	2.628361
1	6.865196	1.000078	2.462581
6	7.277648	-0.466970	3.809002
1	7.694315	0.112489	4.436093
6	6.607325	-2.536212	3.169930
1	6.545481	-3.470401	3.330861
6	4.463634	-8.446834	0.228491
1	4.739940	-9.303411	0.532726
6	3.422882	-8.178499	-0.603027
1	2.844414	-8.830917	-0.979918
6	4.318284	-6.282370	-0.109220
6	6.276458	-7.125882	1.318771
1	6.059999	-6.907926	2.249390
1	6.833844	-6.415413	0.937908
1	6.763805	-7.975627	1.287132
8	-0.274070	-2.196791	1.781674
1	0.157076	-2.574529	1.075358
1	0.321144	-2.210198	2.476012
8	-2.668388	-3.398095	2.020697
1	-1.948609	-3.008564	1.994483
1	-3.088891	-3.091869	2.641911
8	0.991154	-2.877011	-0.563943
1	0.431810	-2.482752	-1.114639
1	0.926502	-3.826371	-0.812690
8	0.959631	-5.490206	-1.460433
1	1.746484	-5.932887	-1.258895
1	1.087482	-5.304840	-2.410508
8	-2.111528	-5.133440	-2.173674
1	-2.006266	-5.344875	-1.163867
1	-1.195171	-5.237310	-2.497147
8	-2.581510	-5.691612	0.470315
1	-3.470475	-5.824476	0.274741
1	-2.552482	-4.986473	1.023029
28	-5.095775	0.828181	0.064164
7	-4.765399	2.794160	0.051744
7	-6.019646	1.402180	-1.726712
7	-5.499081	-1.124277	0.035490

7	-3.383181	0.043693	-0.891604
7	-1.145355	-1.342378	-1.790646
7	-6.917082	0.743666	1.112331
7	-9.353924	0.308073	2.396423
7	-2.081859	1.718936	3.792161
7	-3.901783	1.089067	1.778944
7	-5.304225	-6.149909	-0.186832
7	-7.498635	-5.916823	0.079935
7	-7.038211	2.462459	-4.092226
7	-3.175531	7.521845	0.816182
7	-4.881837	7.940819	-0.545087
6	-4.083773	3.366490	1.056470
6	-3.894050	4.737247	1.104848
1	-3.434586	5.135622	1.834638
6	-4.392235	5.529481	0.057080
6	-5.049153	4.903748	-1.002555
1	-5.362965	5.410929	-1.741970
6	-5.237396	3.530176	-0.960629
6	-5.930740	2.726173	-1.991487
6	-6.633557	-1.572257	0.585839
6	-6.951120	-2.921917	0.587074
1	-7.747981	-3.229684	1.002742
6	-6.078484	-3.824741	-0.034575
6	-4.902178	-3.329545	-0.619018
1	-4.296736	-3.916546	-1.056509
6	-4.638716	-1.973325	-0.547781
6	-2.260143	0.685644	-1.218538
1	-2.224821	1.632648	-1.152046
6	-1.140103	-0.014571	-1.655122
1	-0.347834	0.466750	-1.863189
6	-2.283002	-1.979797	-1.488012
1	-2.325451	-2.922837	-1.594893
6	-3.403404	-1.302900	-1.022378
6	-7.598981	1.750543	1.662413
1	-7.248900	2.632702	1.619085
6	-8.813225	1.525314	2.297091
1	-9.276373	2.264112	2.674396
6	-8.662085	-0.699534	1.848259
1	-9.012399	-1.580883	1.901980
6	-7.445969	-0.495854	1.202348
6	-3.544319	2.370269	2.015699
6	-2.652149	2.668366	3.040131
1	-2.439823	3.578297	3.212281
6	-2.432228	0.457462	3.526436
1	-2.039604	-0.243966	4.032752
6	-3.349671	0.136394	2.536535
1	-3.588646	-0.772169	2.395458
6	-7.233281	-7.257820	0.038537
1	-7.865666	-7.963288	0.108238
6	-5.888245	-7.385924	-0.121465
1	-5.422716	-8.211996	-0.179403
6	-6.300420	-5.273429	-0.054901
6	-8.851150	-5.360292	0.168022
1	-8.923928	-4.583212	-0.424781
1	-9.502595	-6.040235	-0.103422
1	-9.031707	-5.084948	1.091122
6	-6.622360	0.628136	-2.630600
1	-6.710473	-0.303124	-2.464903
6	-7.122993	1.163994	-3.811304
1	-7.539592	0.584465	-4.438415
6	-6.452602	3.233166	-3.172252
1	-6.390894	4.167375	-3.333216
6	-4.308911	9.143788	-0.230813
1	-4.585285	10.000314	-0.535101
6	-3.268226	8.875403	0.600652
1	-2.689759	9.527942	0.977616
6	-4.163629	6.979394	0.106918
6	-6.121803	7.822907	-1.321073
1	-5.905372	7.604901	-2.251610
1	-6.679121	7.112367	-0.940230

1	-6.609083	8.672580	-1.289454

Water cluster with nitro group

8	-2.474490	1.326531	0.000000
8	-2.988690	-0.178869	-1.465900
8	-1.469090	1.301031	-1.923400
7	-2.307790	0.804031	-1.155500
8	-1.271490	3.752231	0.482000
1	-0.760590	4.027431	-0.218500
1	-1.684590	2.984431	0.205600
8	2.882610	6.695031	-4.202800
8	2.177910	8.401431	-3.065300
8	4.281210	8.231031	-3.559000
7	3.131910	7.778731	-3.604700
8	-2.727290	5.855231	1.313300
1	-2.312990	5.184331	1.092500
1	-3.200990	5.654331	1.939200
8	0.665610	4.489431	-1.326200
1	1.160510	4.964031	-0.777200
1	0.378710	5.145131	-2.000800
8	0.090310	6.308031	-3.325000
1	-0.079090	5.889931	-4.132600
1	0.998710	6.635031	-3.470700
8	0.007410	8.592531	-1.124500
1	-0.898790	8.088631	-1.144000
1	0.474110	8.195931	-1.886100
8	-2.573190	7.781331	-0.671100
1	-2.723890	8.555931	-0.198400
1	-2.667690	7.110731	-0.083800
8	-4.516790	5.036331	3.362600
8	-4.002590	6.541731	4.828600
8	-5.522190	5.061831	5.286000
7	-4.683490	5.558931	4.518100

References:

(S1) Rigaku. CrysAlisPro 1.171.42.51a; Rigaku Oxford Diffraction: **2022**.

(S2) Sheldrick, G. SHELXT - Integrated Space-Group and Crystal-Structure Determination. *Acta Crystallogr., Sect. A: Found. Adv*. **2015**, *7*, 3−8.

(S3) Sheldrick, G. M. Crystal Structure Refinement with SHELXL. *Acta Crystallogr., Sect. A: Found. Adv*. **2015**, *71*, 3−8.

(s4) Dolomanov, O. V.; Bourhis, L. J.; Gildea, R. J.; Howard, J. A. K.; Puschmann, H. OLEX2: A Complete Structure Solution, Refinement and Analysis Program. *J. Appl.Crystallogr*. **2009**, *42*, 339−341.
