# Supplementary material for: Comparative Studies on the Structural, Optical, and Electrical Properties of Two [Co(II) and Ni(II)] Complexes: Insights through Theoretical Analysis
Source: J Phys Chem B. 2025 Jul 3;129(28):7389–405. doi: 10.1021/acs.jpcb.5c02577 (PMC12278210; doi:10.1021/acs.jpcb.5c02577)

checkCIF/PLATON report

Structure factors have been supplied for datablock(s) smss_sp_0m_a

THIS REPORT IS FOR GUIDANCE ONLY. IF USED AS PART OF A REVIEW PROCEDURE FOR PUBLICATION, IT SHOULD NOT REPLACE THE EXPERTISE OF AN EXPERIENCED CRYSTALLOGRAPHIC REFEREE.

No syntax errors found. CIF dictionary Interpreting this report

Datablock: smss_sp_0m_a

Bond precision:	C-C = 0.0091 A		Wavelength=0.71073
Cell:	a=9.96(3)	b=13.76(7)	c=14.67(5)
	alpha=95.47(18)	beta=98.52(12)	gamma=101.31(15)
Temperature:	100 K		

	Calculated	Reported
Volume	1934(13)	1932(13)
Space group	P -1	P -1
Hall group	-P 1	-P 1
Moiety formula	C34 H26 Co N14, 2(N O3), 6(H2 O)	C34 H26 Co N14, 2(N O3), 6(H2 O)
Sum formula	C34 H38 Co N16 O12	C34 H38 Co N16 O12
Mr	921.73	921.73
Dx,g cm-3	1.583	1.584
Z	2	2
Mu (mm-1)	0.530	0.531
F000	954.0	954.0
F000'	955.11	
h,k,lmax	12,16,18	12,16,18
Nref	7600	7581
Tmin,Tmax		0.472,0.746
Tmin'		

Correction method= # Reported T Limits: Tmin=0.472 Tmax=0.746
AbsCorr = MULTI-SCAN

Data completeness= 0.998 Theta(max)= 25.999

R(reflections)= 0.0640(5078) wR2(reflections)= 0.1727(7581)

S = 1.030 Npar= 588

The following ALERTS were generated. Each ALERT has the format
test-name_ALERT_alert-type_alert-level.
Click on the hyperlinks for more details of the test.

Alert level B

```
RINTA01_ALERT_3_B  The value of Rint is greater than 0.18
            Rint given   0.213
```

Alert level C

```
PLAT053_ALERT_1_C Minimum Crystal Dimension Missing (or Error) ...     Please Check
PLAT054_ALERT_1_C Medium  Crystal Dimension Missing (or Error) ...     Please Check
PLAT055_ALERT_1_C Maximum Crystal Dimension Missing (or Error) ...     Please Check
PLAT148_ALERT_3_C s.u. on the      a    - Axis is (Too) Large ....      0.030 Ang.
PLAT148_ALERT_3_C s.u. on the      b    - Axis is (Too) Large ....      0.070 Ang.
PLAT148_ALERT_3_C s.u. on the      c    - Axis is (Too) Large ....      0.050 Ang.
PLAT149_ALERT_3_C s.u. on the  alpha    Angle is Too Large ......        0.18 Degree
PLAT149_ALERT_3_C s.u. on the   beta    Angle is Too Large ......        0.12 Degree
PLAT149_ALERT_3_C s.u. on the  gamma    Angle is Too Large ......        0.15 Degree
PLAT341_ALERT_3_C Low Bond Precision on  C-C Bonds ..............    0.00913 Ang.
PLAT906_ALERT_3_C Large K Value in the Analysis of Variance ......      2.257 Check
PLAT911_ALERT_3_C Missing FCF Refl Between Thmin & STh/L=    0.600          9 Report
               -9 11  0,   2 -2  1,   4-10  3,   2 -5  4,   2  1  4,   1 -2  6,
               -2 12  6,   3 -8  7,  -4  9  7,
PLAT975_ALERT_2_C Check Calcd Resid. Dens.  1.05Ang From O63     .       0.55 eA-3
PLAT975_ALERT_2_C Check Calcd Resid. Dens.  1.05Ang From O62     .       0.40 eA-3
```

Alert level G

```
PLAT002_ALERT_2_G Number of Distance or Angle Restraints on AtSite          2 Note
PLAT007_ALERT_5_G Number of Unrefined Donor-H Atoms .............          12 Report
              H58A  H58B  H59A  H59B  H60A  H60B  H61A  H61B  H62A  H62B  H63A
              H63B
PLAT013_ALERT_1_G N.O.K.   _shelx_hkl_checksum Found in CIF ......     Please Check
PLAT020_ALERT_3_G The Value of Rint is Greater Than 0.12 .........      0.213 Report
PLAT172_ALERT_4_G The CIF-Embedded .res File Contains DFIX Records          1 Report
PLAT432_ALERT_2_G Short Inter X...Y Contact  O56      ..C11      .       2.93 Ang.
                                                        x,y,z  =      1_555 Check
PLAT432_ALERT_2_G Short Inter X...Y Contact  O57      ..C7       .       2.99 Ang.
                                                      1+x,y,z  =      1_655 Check
PLAT794_ALERT_5_G Tentative Bond Valency for Co1       (III)     .       3.14 Info
PLAT860_ALERT_3_G Number of Least-Squares Restraints ............           1 Note
PLAT883_ALERT_1_G No Info/Value for _atom_sites_solution_primary .     Please Do !
PLAT910_ALERT_3_G Missing # of FCF Reflection(s) Below Theta(Min).          2 Note
                0  1  0,   0  0  1,
PLAT912_ALERT_4_G Missing # of FCF Reflections Above STh/L=  0.600          4 Note
PLAT933_ALERT_2_G Number of HKL-OMIT Records in Embedded .res File         10 Note
                0  0  1,   0  1  0,   2 -2  1,   2  1  4,  -9 11  0,   4-10  3,
                2 -5  4,  -2 12  6,   3 -8  7,  -4  9  7,
PLAT967_ALERT_5_G Note: Two-Theta Cutoff Value in Embedded .res ..       52.0 Degree
PLAT969_ALERT_5_G The 'Henn et al.' R-Factor-gap value ..........      1.312 Note
              Predicted wR2: Based on SigI**2 13.17 or SHELX Weight 16.77
PLAT978_ALERT_2_G Number C-C Bonds with Positive Residual Density.          1 Info
```

```
 0 ALERT level A = Most likely a serious problem - resolve or explain
 1 ALERT level B = A potentially serious problem, consider carefully
14 ALERT level C = Check. Ensure it is not caused by an omission or oversight
16 ALERT level G = General information/check it is not something unexpected

 5 ALERT type 1 CIF construction/syntax error, inconsistent or missing data
 7 ALERT type 2 Indicator that the structure model may be wrong or deficient
13 ALERT type 3 Indicator that the structure quality may be low
 2 ALERT type 4 Improvement, methodology, query or suggestion
 4 ALERT type 5 Informative message, check
```

It is advisable to attempt to resolve as many as possible of the alerts in all categories. Often the minor alerts point to easily fixed oversights, errors and omissions in your CIF or refinement strategy, so attention to these fine details can be worthwhile. In order to resolve some of the more serious problems it may be necessary to carry out additional measurements or structure refinements. However, the purpose of your study may justify the reported deviations and the more serious of these should normally be commented upon in the discussion or experimental section of a paper or in the "special_details" fields of the CIF. checkCIF was carefully designed to identify outliers and unusual parameters, but every test has its limitations and alerts that are not important in a particular case may appear. Conversely, the absence of alerts does not guarantee there are no aspects of the results needing attention. It is up to the individual to critically assess their own results and, if necessary, seek expert advice.

Publication of your CIF in IUCr journals

A basic structural check has been run on your CIF. These basic checks will be run on all CIFs submitted for publication in IUCr journals (*Acta Crystallographica*, *Journal of Applied Crystallography*, *Journal of Synchrotron Radiation*); however, if you intend to submit to *Acta Crystallographica Section C* or *E* or *IUCrData*, you should make sure that full publication checks are run on the final version of your CIF prior to submission.

Publication of your CIF in other journals

Please refer to the *Notes for Authors* of the relevant journal for any special instructions relating to CIF submission.

PLATON version of 22/08/2024; check.def file version of 21/08/2024

Datablock smss_sp_0m_a - ellipsoid plot

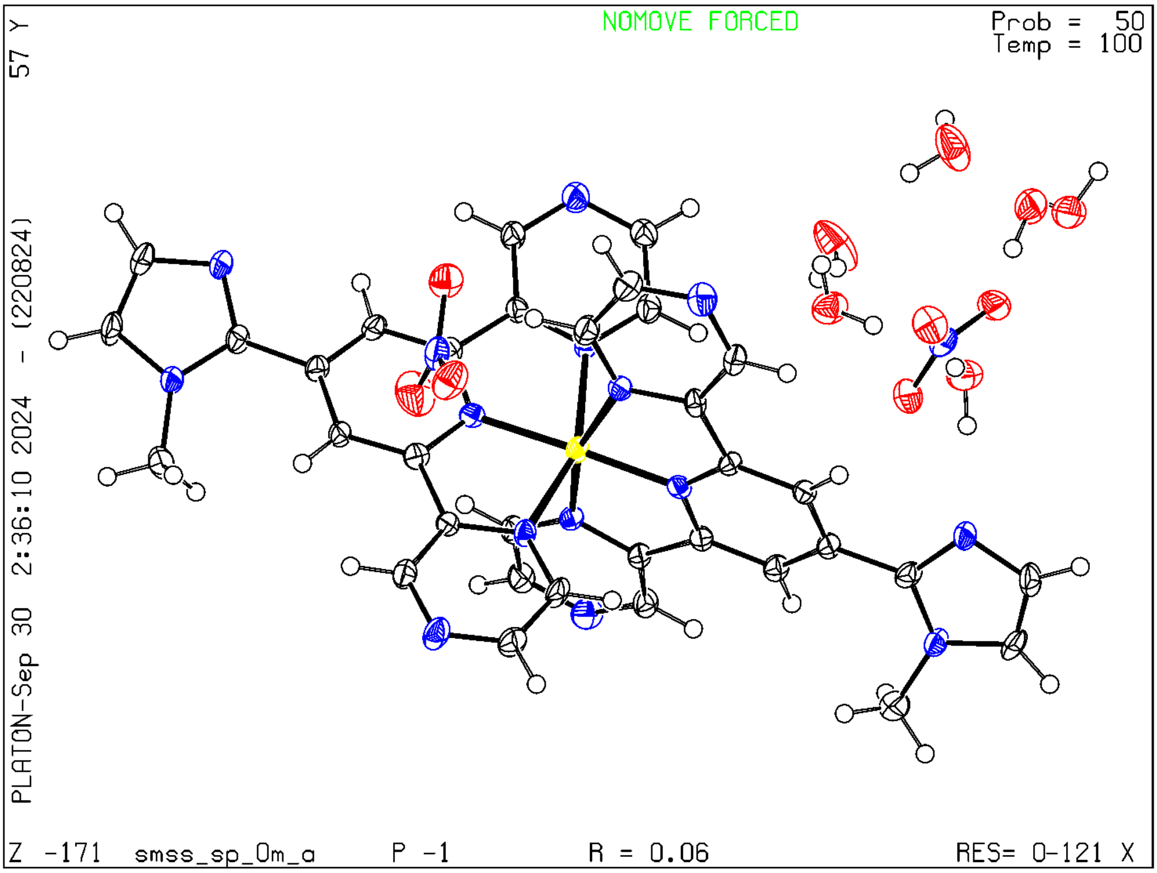

Supplement: Supplementary file 2 [file jp5c02577_si_002.pdf]
