# Supplementary material for: Comparative Studies on the Structural, Optical, and Electrical Properties of Two [Co(II) and Ni(II)] Complexes: Insights through Theoretical Analysis
Source: J Phys Chem B. 2025 Jul 3;129(28):7389–405. doi: 10.1021/acs.jpcb.5c02577 (PMC12278210; doi:10.1021/acs.jpcb.5c02577)

checkCIF/PLATON report

Structure factors have been supplied for datablock(s) 231141lt_auto

THIS REPORT IS FOR GUIDANCE ONLY. IF USED AS PART OF A REVIEW PROCEDURE FOR PUBLICATION, IT SHOULD NOT REPLACE THE EXPERTISE OF AN EXPERIENCED CRYSTALLOGRAPHIC REFEREE.

No syntax errors found. CIF dictionary Interpreting this report

Datablock: 231141lt_auto

Bond precision:	C-C = 0.0019 A		Wavelength=1.54184
Cell:	a=10.1109(2)	b=13.8068(3)	c=14.5968(3)
	alpha=94.968(2)	beta=98.553(2)	gamma=102.076(2)
Temperature:	100 K		

	Calculated	Reported
Volume	1955.79(7)	1955.79(7)
Space group	P -1	P -1
Hall group	-P 1	-P 1
Moiety formula	C34 H26 N14 Ni, 2(N O3), 6(H2 O)	C34 H26 N14 Ni, 2(N O3), 6(H2 O)
Sum formula	C34 H38 N16 Ni O12	C34 H38 N16 Ni O12
Mr	921.49	921.51
Dx,g cm-3	1.565	1.565
Z	2	2
Mu (mm-1)	1.458	1.458
F000	956.0	956.0
F000'	953.14	
h,k,lmax	12,17,18	12,17,17
Nref	8045	7556
Tmin,Tmax	0.758,0.815	0.871,1.000
Tmin'	0.758	

Correction method= # Reported T Limits: Tmin=0.871 Tmax=1.000
AbsCorr = MULTI-SCAN

Data completeness= 0.939 Theta(max)= 74.840

R(reflections)= 0.0292(7191) wR2(reflections)= 0.0731(7556)

S = 1.026 Npar= 619

The following ALERTS were generated. Each ALERT has the format
test-name_ALERT_alert-type_alert-level.
Click on the hyperlinks for more details of the test.

Alert level C

```
PLAT355_ALERT_3_C Long   O-H (X0.82,N0.98A)  O61      - H61A     .       1.04 Ang.
PLAT417_ALERT_2_C Short Inter D-H..H-D       H59A     ..H61A     .       2.11 Ang.
                                                    x,y,z  =      1_555 Check
PLAT911_ALERT_3_C Missing FCF Refl Between Thmin & STh/L=    0.600         53 Report
                2  2  0,   0  1  1, -10  2  3,  -9  4  7,   9  1  8,   9 -3  9,
                9 -2  9,   9 -1  9,   8  0  9,   8  1  9,   8  2  9,   8  3  9,
                8 -2 10,   8 -1 10,   7  0 10,   8  0 10,   7  1 10,   8  1 10,
                7  2 10,   7  3 10,   7  4 10,   8 -6 11,   8 -5 11,   8 -4 11,
                8 -3 11,   8 -2 11,   7 -1 11,   6  0 11,   7  0 11,   7  1 11,
                6  2 11,   7  2 11,  -3 -3 12,  -2 -3 12,   7 -3 12,   7 -2 12,
                6 -1 12,   7 -1 12,   6  0 12,   7  0 12,   6  1 12,   6  2 12,
                6  3 12,   5  5 12,   6 -2 13,   6 -1 13,   6  0 13,   5  1 13,
                6  1 13,  -7 -1 14,   5 -1 14,   5  0 14,   5  1 14,
PLAT975_ALERT_2_C Check Calcd Resid. Dens.  0.83Ang From O62     .       0.42 eA-3
```

Alert level G

```
PLAT154_ALERT_1_G The s.u.'s on the Cell Angles are Equal ..(Note)      0.002 Degree
PLAT232_ALERT_2_G Hirshfeld Test Diff (M-X)  Ni1      --N2       .         5.6 s.u.
PLAT232_ALERT_2_G Hirshfeld Test Diff (M-X)  Ni1      --N9       .         6.2 s.u.
PLAT432_ALERT_2_G Short Inter X...Y Contact  O56      ..C11      .        2.93 Ang.
                                                 -1+x,y,z  =      1_455 Check
PLAT794_ALERT_5_G Tentative Bond Valency for Ni1       (II)      .        2.25 Info
PLAT912_ALERT_4_G Missing # of FCF Reflections Above STh/L=  0.600         415 Note
PLAT913_ALERT_3_G Missing # of Very Strong Reflections in FCF ....           1 Note
                2  2  0,
PLAT941_ALERT_3_G Average HKL Measurement Multiplicity ..........         3.1 Low
PLAT978_ALERT_2_G Number C-C Bonds with Positive Residual Density.         13 Info
```

0 **ALERT level A** = Most likely a serious problem - resolve or explain
0 **ALERT level B** = A potentially serious problem, consider carefully
4 **ALERT level C** = Check. Ensure it is not caused by an omission or oversight
9 **ALERT level G** = General information/check it is not something unexpected

1 ALERT type 1 CIF construction/syntax error, inconsistent or missing data
6 ALERT type 2 Indicator that the structure model may be wrong or deficient
4 ALERT type 3 Indicator that the structure quality may be low
1 ALERT type 4 Improvement, methodology, query or suggestion
1 ALERT type 5 Informative message, check

It is advisable to attempt to resolve as many as possible of the alerts in all categories. Often the minor alerts point to easily fixed oversights, errors and omissions in your CIF or refinement strategy, so attention to these fine details can be worthwhile. In order to resolve some of the more serious problems it may be necessary to carry out additional measurements or structure refinements. However, the purpose of your study may justify the reported deviations and the more serious of these should normally be commented upon in the discussion or experimental section of a paper or in the "special_details" fields of the CIF. checkCIF was carefully designed to identify outliers and unusual parameters, but every test has its limitations and alerts that are not important in a particular case may appear. Conversely, the absence of alerts does not guarantee there are no aspects of the results needing attention. It is up to the individual to critically assess their own results and, if necessary, seek expert advice.

Publication of your CIF in IUCr journals

A basic structural check has been run on your CIF. These basic checks will be run on all CIFs submitted for publication in IUCr journals (*Acta Crystallographica*, *Journal of Applied Crystallography*, *Journal of Synchrotron Radiation*); however, if you intend to submit to *Acta Crystallographica Section C* or *E* or *IUCrData*, you should make sure that full publication checks are run on the final version of your CIF prior to submission.

Publication of your CIF in other journals

Please refer to the *Notes for Authors* of the relevant journal for any special instructions relating to CIF submission.

PLATON version of 29/11/2023; check.def file version of 14/09/2023

Datablock 231141lt_auto - ellipsoid plot

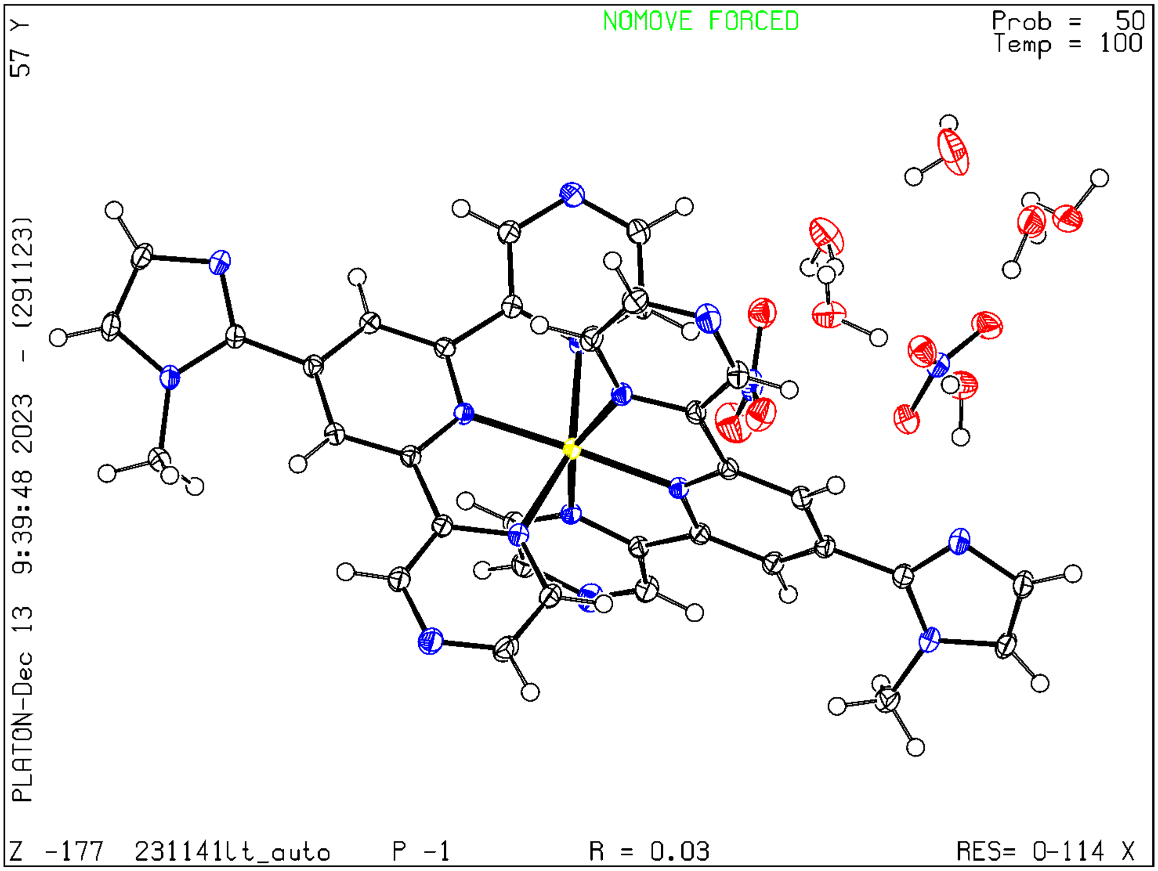

Supplement: Supplementary file 3 [file jp5c02577_si_003.pdf]
